# Protein Languages Differ Depending on Microorganism Lifestyle

**DOI:** 10.1371/journal.pone.0096910

**Published:** 2014-05-14

**Authors:** Joseph J. Grzymski, Adam G. Marsh

**Affiliations:** 1 Division of Earth and Ecosystem Sciences, Desert Research Institute, Reno, Nevada, United States of America; 2 Center for Bioinformatics and Computational Biology, Marine Biological Sciences, University of Delaware, Lewes, Delaware, United States of America; The Centre for Research and Technology, Hellas, Greece

## Abstract

Few quantitative measures of genome architecture or organization exist to support assumptions of differences between microorganisms that are broadly defined as being free-living or pathogenic. General principles about complete proteomes exist for codon usage, amino acid biases and essential or core genes. Genome-wide shifts in amino acid usage between free-living and pathogenic microorganisms result in fundamental differences in the complexity of their respective proteomes that are size and gene content independent. These differences are evident across broad phylogenetic groups–a result of environmental factors and population genetic forces rather than phylogenetic distance. A novel comparative analysis of amino acid usage–utilizing linguistic analyses of word frequency in language and text–identified a global pattern of higher peptide word repetition in 376 free-living versus 421 pathogen genomes across broad ranges of genome size, G+C content and phylogenetic ancestry. This imprint of repetitive word usage indicates free-living microorganisms have a bias for repetitive sequence usage compared to pathogens. These findings quantify fundamental differences in microbial genomes relative to life-history function.

## Introduction

Microorganisms exhibit a wide range of environmental adaptations and lifestyles encoded by their genomes [1]–[5]. Our understanding of the limits of microbial life on Earth keep expanding as microbes are found in myriad, unique environments [6], [7] and as synthetic biology has developed [8], [9] to explore the minimum gene sets required for life [10]–[12]. Progress in both fields, however, is limited by lack of understanding of the genomic rule set or principles that shape gene structure and organization for either life in a specific habitat (e.g., hydrothermal vent, metazoan host, industrial bioreactor) or a defined life-history strategy (e.g., chemoautotrophy, heterotrophy, methanotrophy). Pathogens containing nearly minimal gene sets needed to survive in a host are generally considered to have smaller genome sizes and less complexity than free-living organisms [13], [14]. Genome size, however, is merely a consequence of net gene loss (or gain); it cannot be used to distinguish free-living organisms from pathogens because of the broad overlap in genome sizes that exist between these two groups. Even within a broad group defined as “pathogen”, there is a range of life histories. Furthermore, recent analyses and single-cell amplified genome sequencing revealed that many oligotrophic marine microbes are cost-minimized and have small, low GC genomes [15], [16]. Genome streamlining [17] appears to be an important feature of free-living marine oligotrophic microbes [16].

Genomes are highly organized information structures [18]. Working with sequence entropy is one way to formulate information or organization in whole genome sequences [19]–[21]. A high level of local sequence organization can be assessed with bibliometrics where large differences in information structure are evident among different genomes [22], [23]. Local sequence organization in the form of multiple alignments of amino acid blocks or short motifs has been used in protein classification for two decades [24]. An extension of this concept is maximum entropy models which have been used to characterize sequence diversity in antibodies and provide a mathematical framework for extracting quantitative information from experimental data [25]. As well, heuristic models from large environmental data sets are being used to relate genomic information to trophic lifestyle [15], [26]. We focused on isolating and characterizing information content as a way to more fully understand how local amino acid sequence features can be exploited further to provide functional information about unknown or poorly characterized open reading frames (ORFs). There is a pressing need for analytical tools to extract as much information as possible from all currently available genome sequences – not just well-annotated genes.

We hypothesized that any of the evolutionary bottlenecks that occur in obligate/facultative intracellular organisms (e.g., [27]) should impact the entire proteome and alter genome-wide patterns of amino acid word usage. These patterns should be evident in the broad group of organisms defined as pathogens. The goal of this analysis was to establish rule sets or pattern principles to describe genome-level differences between free-living and pathogenic bacteria arising from the major shift in gene function associated with their ecology and evolution. Our results illustrate fundamental differences in the genome architecture of free-living and pathogen genomes, independent of genome size, G+C content or phylogenetic ancestry. This approach perhaps can be exploited to reveal new information about pathogens and our attempts to control them.

## Results and Discussion

We analyzed amino acid word usage in the predicted proteomes of 797 genomes from two categories of microorganisms: free-living microbes (marine and/or terrestrial) and known pathogens (obligate or facultative; Table S1). These categories were based on keyword filters applied to National Center for Biotechnology Information (NCBI) genome submission data. The definitions “free-living” and “pathogen” have broad meanings, and this breadth increases the variance that must be isolated in analyses, not the fundamental differences underlying these categories. For the remainder of the discussion, we refer to these groups as FREE and PATH with the understanding that many pathogens during their lifecycle are not obligately associated with a host.

Our strategy was derived from linguistic analyses of word frequency in language and text [21], [28]. The predicted proteome of each genome was first pre-processed to remove duplicate or redundant proteins greater than 95% identical in sequence. This non-redundant proteome of each genome was broken into “words” from two-to-twelve amino acids long. Observed and expected frequencies of these words within a genome were compiled into reference dictionaries for data retrieval during analysis. To eliminate confounding effects of genome size and G+C content and to explore the importance of phylogenetic grouping, analyses were repeated on randomized copies of the genomes by shuffling all proteome amino acids as one large sequence string and then dividing back to the original ORF number and sizes.

**Figure 1:**
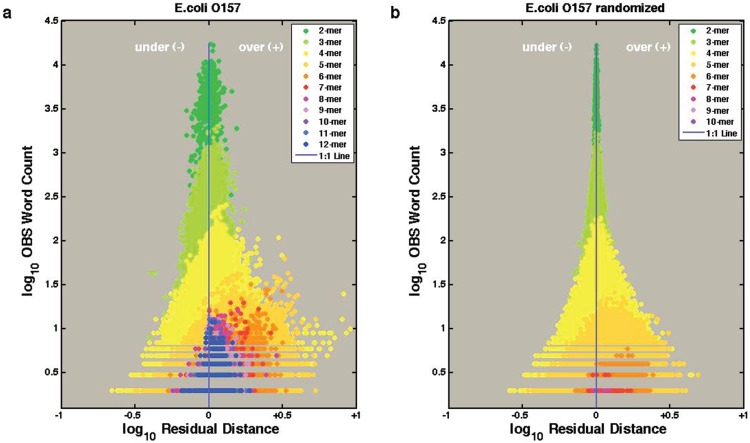
E. coli O157 amino acid dictionaries. Over- and underrepresentation of repetitive amino acid words is plotted for E. coli O157 as the residual difference between Observed and Expected counts of each word (from 2 to 12 mers). (a) Word counts of the non-redundant (cdhit 95%), protein-coding genes of the native E. coli O157 genome (n = 555753 repeated amino acid words); (b) Word counts after randomizing the amino acid sequence of the non-redundant, protein-coding genes of E. coli O157 (n = 433566 repeated amino acid words).

The amino acid word dictionary of a genome contains frequency counts of all N mer amino acid words present in non-redundant predicted proteins. Knowing counts for any N mer length, it is trivial to calculate expected frequency of any N+1 mer in a neutral (null) recombination distribution. For example, in an organism that uses alanine 5%, the frequency of a homodipeptide AA is 0.25%. A focus of this informatic method is to provide a statistical measure to identify motifs that are weak links in the proteome of a pathogen. Targeting these weak links could have a significant impact on pathogen survival (fitness). We assessed the severity of the retention or overrepresentation of specific words within proteins by a statistical analysis looking at amino acid word usage patterns that are in disequilibrium with the usage expected in a null selection model. In Figure 1a, observed and expected word counts for E. coli O157 evidence a skew toward over-representation (above expected values) of many amino acid words of 5 to 12 residues. By comparison, a randomized O157 genome (same amino acid usage and protein number and length; Figure 1b) shows far smaller differences between observed and expected counts, and far fewer words longer than five mers that are repeated after randomization.

**Figure 2:**
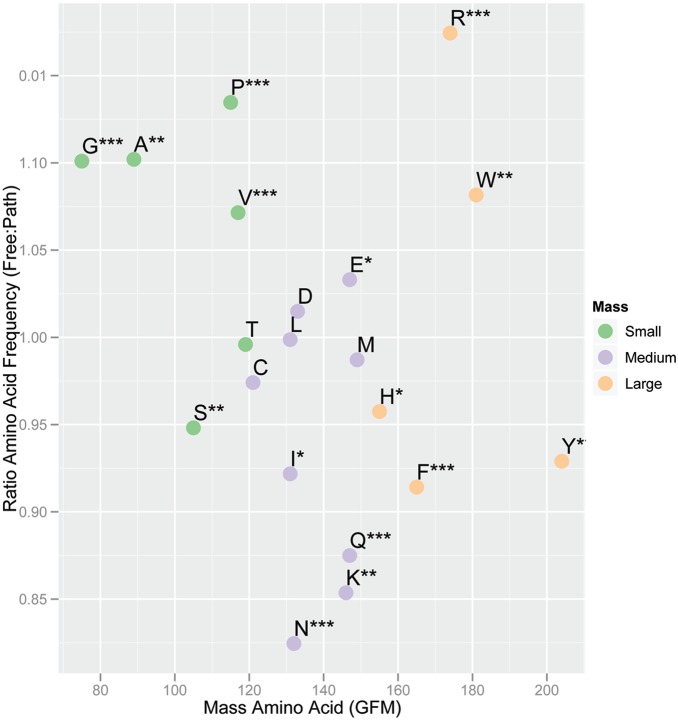
Ratio of free-living and pathogen amino acid usage versus amino acid mass. Data were plotted from the values and statistics presented in Table 1.

Obviously, genomes are not random collections of amino acids, but the striking difference between the two panels in Figure 1 illustrates how the complexity of natural genomes can be measured in terms of overrepresentation or repetition of key amino acid words (peptide motifs). These words likely form local domains in proteins such that a singular amino acid combination is more likely to be successful as a sequence unit within a protein than other possible variants. This is a direct result of natural selection favoring retention or co-evolution of functional/structural sequence blocks [29]. As well, overrepresentation of non-functional sequence blocks could be the result of genetic drift, codon bias, or other random effects. The departure between word-observed counts and neutral expected counts thus can be considered an index of these forces driving retention or maintenance of a word across many genes within a genome. These values are difficult to compare among genomes, however, because of differences in amino acid word usage. Even single amino acid frequencies can be highly variable (Table 1; Figure 2) [30]–[32]. Despite the large number and diverse genomes in this analysis, the majority of amino acids that occur in statistically significant higher frequency in PATH are greater than 130 gram formula mass (GFM) with the exception of arginine and tryptophan which are found in higher frequency in the FREE data set. The two smallest amino acids, glycine and alanine, are found in statistically higher frequency in the FREE data set despite the broad range of data (Table S1). Cost minimization requirements for FREE organisms are not as necessary in PATH [30], [33]. Our method and analysis extend this argument by quantifying a metric of the complexity of higher order amino acid word usage.

**Table 1:** Comparison of amino acid frequencies in all annotated proteins among free-living (Free) and pathogenic (Path) microbes.

Amino Acid	GFM	FREE (mean frequency)	PATH (mean frequency)	p-value
A	89	0.0948	0.0860	2.060e-05
C	121	0.0094	0.0097	NS
D	133	0.0539	0.0531	NS
E	147	0.0635	0.0614	2.833e-03
F	165	0.0396	0.0434	1.035e-11
G	75	0.0756	0.0686	2.263e-16
H	155	0.0199	0.0208	2.624e-04
I	131	0.0647	0.0702	2.213e-04
K	146	0.0515	0.0604	7.478e-07
L	131	0.1021	0.1023	NS
M	149	0.0237	0.0240	NS
N	132	0.0373	0.0452	1.511e-12
P	115	0.0453	0.0399	2.675e-14
Q	147	0.0341	0.0389	1.220e-13
R	174	0.0576	0.0490	1.103e-12
S	105	0.0589	0.0621	1.061e-08
T	119	0.0526	0.0529	NS
V	117	0.0725	0.0676	1.375e-12
W	181	0.0120	0.0110	1.883e-05
Y	204	0.0301	0.0324	2.772e-05

A Welsh’s two-sample t-test was used to compare the mean frequencies and test for the likelihood that the difference among Free and Path observations was not zero. This statistic essentially establishes a 95% confidence interval around the difference of means and assigns significance based on how far the observed arithmetic difference is from 0.

**Figure 3:**
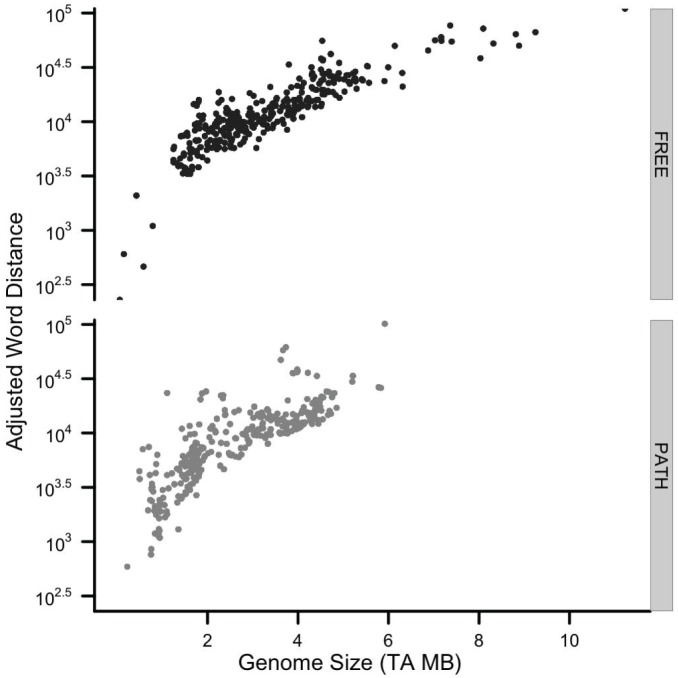
Residual word distance of free-living and pathogen genomes. Total word distance minus the random dictionary contribution (see Figure 4) plotted as a function of genome size.

The observed-minus-expected residual distance of amino acid words among 376 FREE and 421 PATH genomes differs across a broad range of phylogeny, genome size and % G+C content (see Table S1). In Figure 3, residual distances (adjusted for variation present in the randomized copy of each genome by subtraction) were plotted against genome size (calculated from the non-redundant, protein-coding regions). We found a strong relationship between size and the adjusted word distance with larger genomes utilizing higher amino acid word repetition. But the opposite trend is just as intriguing – as size decreases, there appears to be a genome minimum around 0.5 MB where the sum of the differences between observed and expected word counts would be the same as the residual distance found in their randomized versions. Although the limit in this plot has a wide confidence interval, it raises two timely questions: 1) what are the smallest free-living vs. pathogen genome sizes possible? and 2) what is it about amino acid word usage that impacts gene composition to determine those size limits?

**Figure 4:**
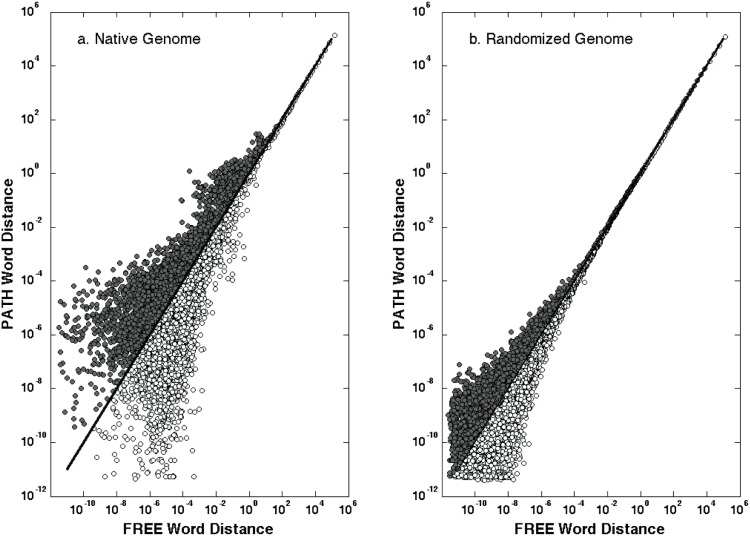
Residual word distance of native and random genomes. Residual word distances for individual genomes in free-living (n = 376) and human-pathogen (n = 421) microbes were divided into class levels based on the number of times words were repeated within a genome, from 2 to 30,000. A group mean of the word distance at each repeat level was calculated and plotted above as FREE vs. PATH for the native genome data set (a) and the randomized genome data set (b).

In order to compare total word utilization patterns among FREE and PATH genomes, we reduced the 2-to-12 mer amino acid word dictionaries of each genome and an identical, randomized copy to a 30,000 element vector with each i^th^ element representing total residual distance between observed and expected counts in that dictionary for all words repeated i times. This finite vector condensed amino acid word dictionaries into a numerical array directly comparable among genomes. Here, the sum of the observed minus expected deviations in amino acid words repeated between 2 and 30,000 times is independent of either the length of those words or their specific amino acid sequence. We described the degree to which some local domain sequences were retained across many genes within a genome by comparing distributions of these word counts. The fundamental differences between the two groups are highlighted in a comparison plot of these data for native and randomized genomes (Figure 4). This phenomenon is not a function of genome size, localized regions in a genome, or phylogeny. If it were, then the native and random plots would not differ significantly. Furthermore, there would be no evidence of difference in the native genomes of FREE versus PATH (Figure 4a). The asymmetric distribution of word distance where the PATH repeat bin is greater than free-living organisms (closed circles) or vice versa (open circles) suggests fundamental differences in word usage architecture among the groups. These differences were subsequently analyzed using a series of statistical tests.

In comparing word distance among genomes after size normalization, differences in word repeat distributions at a global level could be a function of organism lifestyle. That is, there is some global selection pressure, for example, to reduce GC content and streamline the genome as an adaptive mechanism to thrive in an environment like the oligotrophic ocean [15], [16], [26] or in obligate intracellular organisms (Figure 4a) [13], [27]. These distributions are not evident in the respective randomized genomes (Figure 4b). Word repeat distributions also could arise from gene duplications, deletions, recombination, point mutation, horizontal transfer, and random genetic drift. Regardless, our results suggest that there are quantifiable differences in the representation of amino acid words between FREE and PATH genomes that have appeared during their evolution.

**Figure 5:**
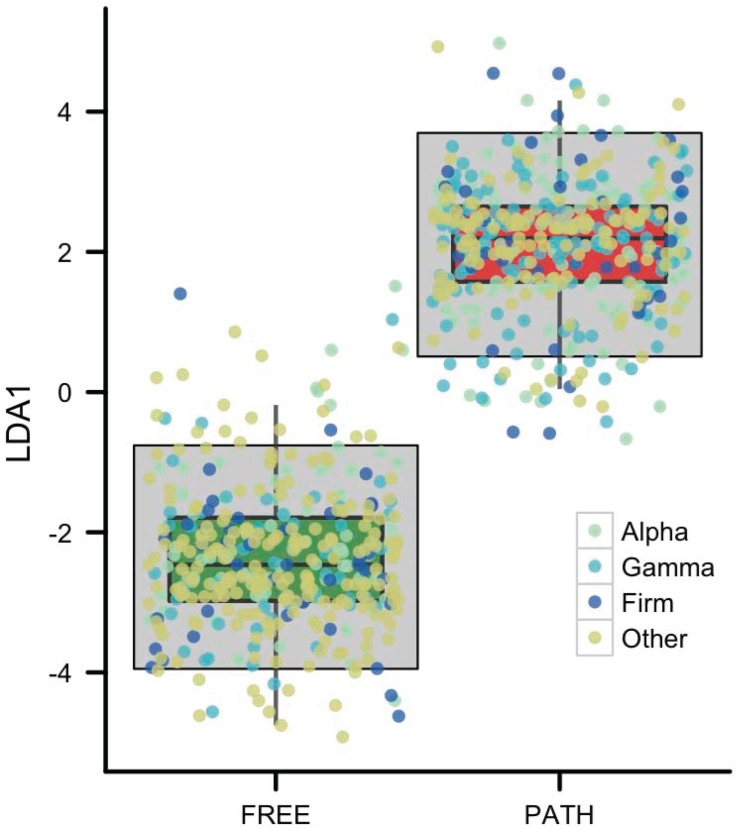
LDA plot with color-coded phylogenetic groups. Linear discriminant analysis of the repeat bin word distance results (Figure 4) between free-living and pathogen genomes. The gray box represents statistical significance (p<10^–6^). The points of genomes from the three largest phylogenetic groups in the data set are highlighted to show no phylogenetic significance of differences in groups.

**Figure 6:**
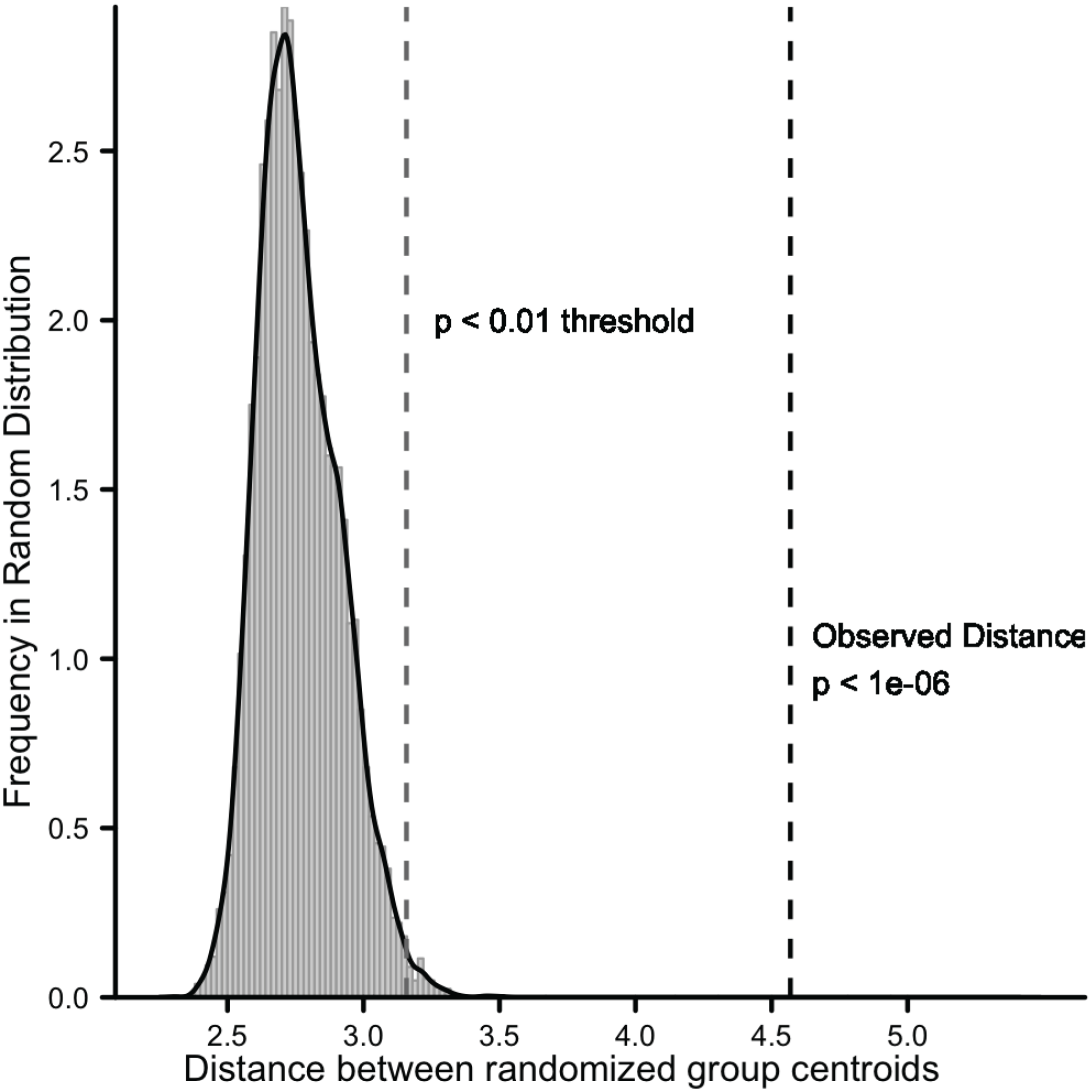
Monte Carlo results for the separation distance between group means in the MDS-LDA analysis of residual word distance between free-living and pathogenic bacteria. Plot shows a frequency distribution for the mean separation between groups over 10 = 0.01 and p = 1e^–06^ boundaries are indicated.

We employed multidimensional scaling analysis on word distance vectors coupled with a linear discriminant function analysis [34]. This enabled us to assess differences in amino acid word usage patterns among individual genomes in the FREE and PATH groups (Figure 5). We utilized this test because of its sensitivity in detecting group-level structures or patterns where group identities are known already. We used a Monte Carlo permutation test on the distance between group centroids to determine random probability of the observed separation between group centroids (Figure 6). Separation among individual genomes into FREE and PATH distributions along the LDA axis was highly significant (p<10^–6^ indicated by the gray box). The group mean differences in Figure 5 indicate that FREE and PATH amino acid word usage patterns are fundamentally different and can be used to characterize the groups. These differences are not merely a function of differences in amino acid composition, genome size or G+C content because they are absent in each randomized genome where these parameters are preserved. Furthermore, the impact of phylogenetic ancestry on the analysis is minimal. In Figure 5, we highlighted the FREE and PATH genomes from the largest three groups [Alphaproteobacteria (n = 119), Gammaproteobacteria (n = 237) and Firmicutes (n = 206)]. Phylogenetic group identity of each genome is color coded, and we see that despite broad phylogenetic differences among these genomes, there is no coherent expression of a phylogenetic signal between FREE and PATH functional groups.

The significance of these findings is that, through time, specific sequence blocks may be preferentially retained in a genome among heterologous genes through any of a variety of mechanisms (Figure 1) as has been recently shown with experimental data [29]. Retention of these redundant motifs is a hallmark of free-living genomes and allows us to differentiate these genomes from pathogen genomes (Figures 5 and 6). On a global level, across an entire genome, our results suggest that repeat elements in a genome may be retained more frequently in highly interactive environments such as soil or ocean microbiomes, and that in such dynamic environments, genomes evolve with increasing complexity or order. Motif diversity decreases and the frequency of preferential motifs increases in dynamic environments. For example, organisms well-adapted to a copiotrophic (high-nutrient), dynamic environment have distinct genomic features compared to organisms well-adapted to low-nutrient, almost steady-state environments [26]. Especially in single celled free-living organisms, we think a more accurate model of genome architecture that accounts for both fitness and genotypic diversity is based on the modular or motif-driven nature of genes and proteins.

The persistent repetition of amino acid words in free-living organisms is significantly greater than in pathogens (Figure 3). The higher repetition of words in the genomes of free-living organisms than in the genomes of pathogens indicates that, in comparison, free-living microbes appear to be subjected to greater functional and structural constraints on their proteins than pathogens. While the relative simplicity of life as a pathogen has been suggested [10], our results provide a quantitative and statistically robust analysis of differences in genome structure (complexity) and suggest that a first principle of genome architecture is a fundamental sequence bias toward redundant amino acid motifs and domains (word-sequence building blocks). This reveals a mechanistic constraint on genomes in organisms that have specific lifestyles (free-living) and tolerate specific environmental conditions (e.g., high temperature) as has been recently shown for marine microbes that live in high- and low-nutrient waters [15], [26].

**Figure 7:**
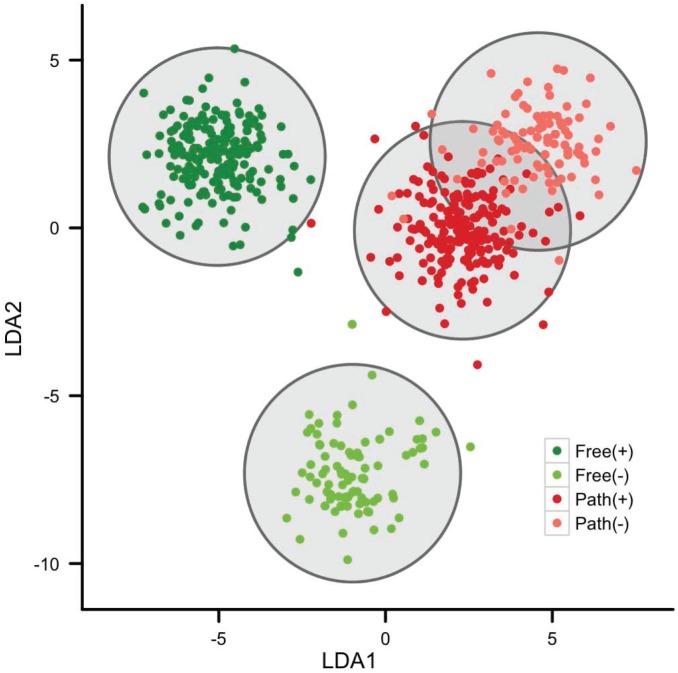
Non-metric multidimensional scaling analysis of amino acid word usage in microbial genomes divided by lifestyle (free-living vs. pathogenic) and gram-staining (+ vs −). A linear discriminate analysis of the MDS coordinates was utilized for Monte Carlo, bootstrap iterations (10,000) of the separation among group centroids when observations are randomly distributed among groups. Probability values indicate the likelihood that the observed centroid separation could arise by random chance alone, and the (p<1e-06) values are indicated by the gray circles.

Analysis of amino acid word usage patterns can delineate more refined functional groupings than just free-living vs. pathogenic microbes. If environmental communication is an important selection force differentiating free-living from pathogen microbes, then we expect cell wall structure, biosynthesis and signaling mechanisms to contribute toward overall fitness. Figure 7 presents the further separation of free-living and pathogen bacteria into gram positive and negative groups. There is remarkable separation between free-living gram positive and negative groups compared to each other and both groups of pathogens. Separation among the gram positive and negative pathogens is less distinct. Metrics of how word sequences are utilized within a genome may be able to capture differences in higher-level fitness functions such as cell to environment communication, or at least analyses such as this may establish relevant hypotheses for further pursuit and validation. In Figure 5, it is intriguing to ask if the selective value of a cell wall is more positive (or negative) for free-living organisms compared to pathogens. Forces of host and self-recognition may be common evolutionary drivers across broad groups of pathogens. Delving into word usage patterns among cell wall proteins, signal receptors and signal transduction could be a fruitful informatic approach to further understand this delineation.

As an example of the power of examining deviations in word usage, and using this technique to better define the architecture of broad groups of organisms, we compared the shared amino acid six-mer words between gram-positive free-living and gram-positive pathogenic microbes. We calculated the average deviation of a six-mer word’s expected probability from its observed frequency in any genome and averaged across each genome in a group. The top ten words shared in common with the greatest deviation in occurrence between gram-positive pathogen and gram-positive free-living organisms are presented in Table 2. These motifs that are either retained more in pathogens or in free-living gram positive genomes point to proteins that can be used to understand differences in the groups. For example, the motif DLAGIG was found far more frequently than expected in pathogen gram-positive genomes. This motif is found in UDP-N-acetylmuramoyl-L-alanyl-D-glutamate synthetase – an important contributor to cell-wall synthesis. Mutations in this protein confer different resistances to cell-wall targeted antibiotics in gram-positive organisms [35], [36]. This observation encompasses a broad set of genomes. We have strong quantitative evidence that a DLAGIG word in enzymes involved with polysaccharide synthesis is significant in gram-positive pathogens. Thus, with this approach, we can link specific amino acid words to specific proteins and then to very specific, functional selection pressures. This information is vital to developing potentially new ways to target pathogens – especially those that are currently drug or multi-drug resistant.

**Table 2:** Comparison of the shared amino acid 6-mer words with the greatest average sequence score among gram-positive free-living (Free) and pathogenic (Path) microbes.

Rank	Word	Score	CDD^1^	DB^2^	Description	E-value
PATHOGENS						
1	DLAGIG	373.6	100866	PRK01390	UDP-N-acetylmuramoyl-L-alanyl-D-glutamatesynthetase	26
2	PLADLL	255.0	118395	pfam09865	Predicted periplasmicprotein (DUF2092)	14
3	SGLGLY	246.6	115888	pfam07262	Protein of unknown function (DUF1436).This family consists of several hypotheticalbacterial proteins	19
4	IPVDGE	241.4	88415	cd05798	Transaldolase (TAL)/Phosphoglucose isomerase (PGI);Involved with the themicrobial conversion of D-arabitolto xylitol	7.9
5	IRDDLI	232.5	102253	PRK06207	Aspartate aminotransferase	4.4
6	MILLGI	228.3	110765	pfam01790	Prolipoprotein diacylglyceryl transferase	4.4
7	KQALKD	226.4	107063	PHA01750	Hypothetical protein	14
8	TVTADR	211.7	115489	pfam06835	Protein of unknown function (DUF1239).This family consists of several hypotheticalbacterial proteins	14
9	RINELA	208.0	101064	PRK02539	Hypothetical protein	7.9
10	GHPDVF	204.6	31444	COG1252	NADH dehydrogenase, FAD-containing subunit	19
**FREE-LIVING**	****					
1	TYAELD	437.3	103683	PRK09088	Acyl-CoA synthetase	5.9
2	GVLPRT	307.6	105426	PRK11824	Polynucleotide phosphorylase/polyadenylase	14
3	GASGFL	295.7	106095	PRK13114	Tryptophan synthase subunit alpha	34
4	PLSPAQ	295.0	112395	pfam03576	Peptidase family S58	14
5	DRPRPA	283.8	105673	PRK12467	Peptide synthase	5.9
6	IDTATN	271.4	33198	COG3391	Uncharacterized conserved protein[function unknown]	11
7	AAPPPP	257.8	115804	pfam07174	Bacterial fibronectin-attachmentprotein (FAP)	11
8	GTPVAG	254.3	104702	PRK10644	Arginine: agmatin antiporter	34
9	IAAGEK	244.1	103529	PRK08654	Pyruvate carboxylase subunit A	26
10	FSGGEK	240.9	104694	PRK10636	Putative ABC transporter ATP-binding protein	19

NOTE: The gram-positive FREE and PATH dictionaries used in the LDA analysis for Fig. 4 were merged into an “averaged” dictionary of 6-mer amino acid words that were present in both groups. The common 6-mer words with the largest difference (expressed as a ratio) in selection scores between FREE and PATH word distance were aligned against NCBI’s Conserved Domain Database to identify potential proteins in which these words appear.

(1) Conserved Domain Database: http://www.ncbi.nlm.nih.gov/Structure/cdd/cdd.shtml.

(2) Cross-referenced database entry within CDD.

**Figure 8:**
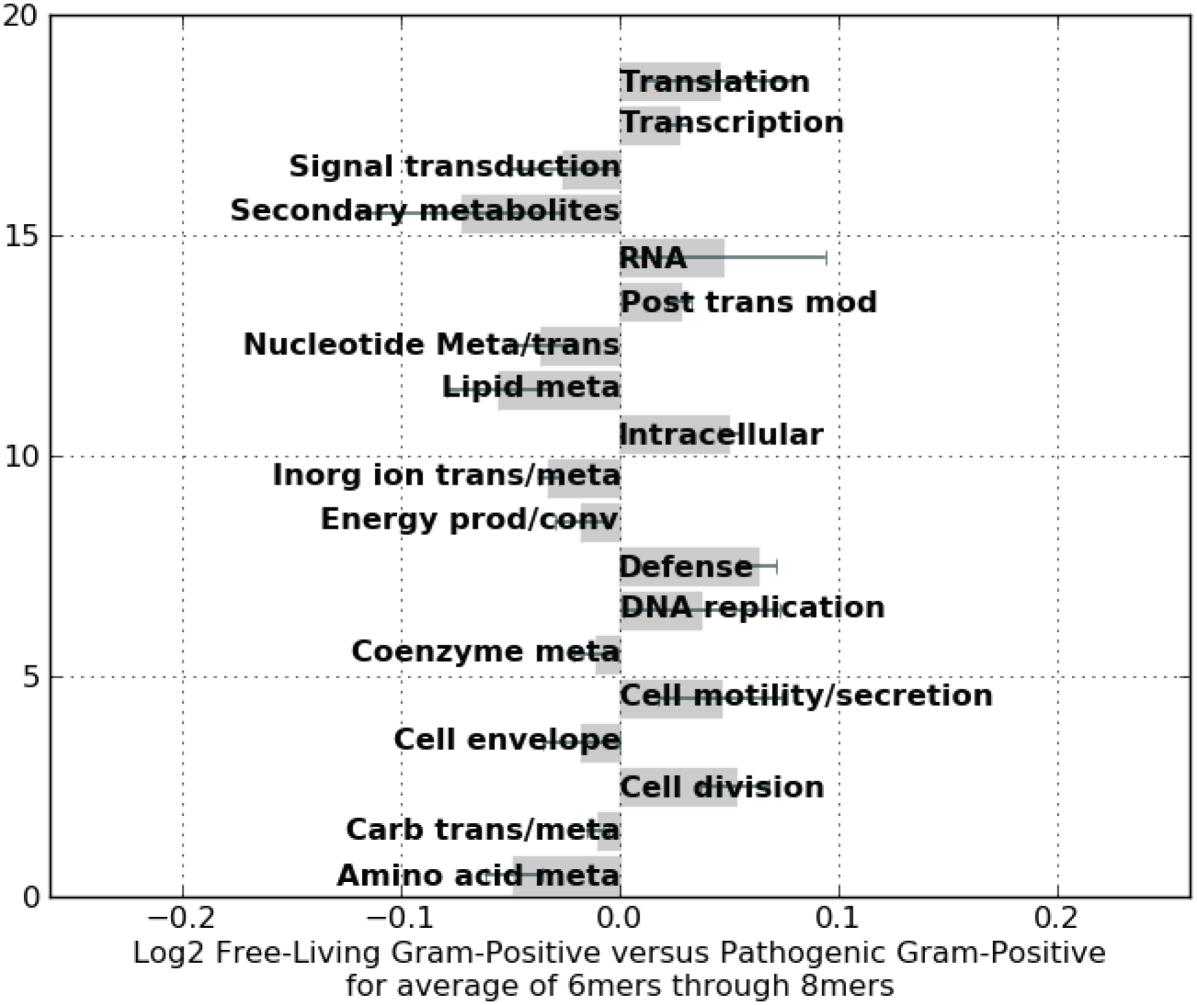
Distribution of overrepresented words (averaged 6–8 mers) in free-living gram-positive compared to pathogenic gram-positive bacteria. Each bar represents words overrepresented in free-living (negative) or pathogenic (positive) gram-positive genomes. Genomes utilized in the analysis are listed in Table 3.

Likewise, these motif statistics can be accumulated for select groups of genomes for comparison. Figure 8 shows COG functional category differences in the cumulative 6–8 mer motifs that are most overrepresented between a group of 42 gram-positive pathogens and a group of 42 gram-positive free-living bacteria (Table 3). Here, overrepresented or highly selected motifs appear more often in defense, intracellular and cell division related proteins in gram-positive pathogens compared to proteins in gram-positive free-living bacteria. Highly selected motifs in gram-positive free-living bacteria are found in amino acid and secondary metabolite biosynthesis. Both observations suggest specific hypotheses for further experimental validation based on metabolic cost differences between the two groups and the constant need of pathogens to defend against host immune response.

**Table 3:** List of organisms used in the analysis presented in Figure 8.

Free-living gram positive organisms	GenBank PID
Acidothermus cellulolyticus 11B	16097
Anoxybacillus flavithermus WK1	28245
Arthrobacter aurescens TC1	12512
Arthrobacter chlorophenolicus A6	20011
Candidatus Desulforudis audaxviator MP104C	21047
Clostridium acetobutylicum	77
Clostridium cellulolyticum H10	17419
Clostridium novyi NT	16820
Clostridium thermocellum ATCC 27405	314
Corynebacterium efficiens YS-314	305
Corynebacterium glutamicum R	19193
Corynebacterium jeikeium K411	13967
Dehalococcoides BAV1	15770
Dehalococcoides ethenogenes 195	214
Deinococcus geothermalis DSM 11300	13423
Deinococcus radiodurans	65
Dictyoglomus turgidum DSM 6724	29175
Geobacillus kaustophilus HTA426	13233
Geobacillus thermodenitrificans NG80-2	18655
Lactobacillus acidophilus NCFM	82
Lactobacillus delbrueckii bulgaricus	16871
Lactobacillus fermentum IFO 3956	18979
Lactobacillus sakei 23K	13435
Listeria innocua	86
Listeria welshimeri serovar 6b SLCC5334	13443
Mycobacterium JLS	16079
Mycobacterium KMS	16081
Salinispora arenicola CNS-205	17109
Salinispora tropica CNB-440	16342
Streptomyces avermitilis	189
Streptomyces coelicolor	242
Streptomyces griseus NBRC 13350	20085
Symbiobacterium thermophilum IAM14863	12994
Thermoanaerobacter pseudethanolicus ATCC 33223	13901

Pathogenic gram positive organisms	GenBank PID
Bacillus anthracis Ames 0581	10784
Bacillus cereus B4264	17731
Bacillus thuringiensis Al Hakam	18255
Bacillus weihenstephanensis KBAB4	13623
Bacteroides vulgatus ATCC 8482	13378
Clavibacter michiganensis NCPPB 382	19643
Clostridium botulinum E3 Alaska E43	28855
Clostridium difficile 630	78
Clostridium perfringens	79
Clostridium tetani E88	81
Corynebacterium diphtheriae	87
Corynebacterium urealyticum DSM 7109	29211
Enterococcus faecalis V583	70
Listeria monocytogenes HCC23	29409
Lysinibacillus sphaericus C3 41	19619
Mycobacterium abscessus ATCC 19977T	15691
Mycobacterium bovis	89
Mycobacterium marinum M	16725
Mycobacterium smegmatis MC2-155	92
Parabacteroides distasonis ATCC 8503	13485
Propionibacterium acnes KPA171202	12460
Renibacterium salmoninarum ATCC 33209	19227
Staphylococcus aureus RF122	63
Staphylococcus eermidis RP62A	64
Staphylococcus haemolyticus	12508
Staphylococcus saprophyticus	15596
Streptococcus gordonii Challis substr CH1	66
Streptococcus sanguinis SK36	13942
Streptococcus suis 98HAH33	17155
Streptococcus uberis 0140J	353
Thermobifida fusca YX	94
Tropheryma whipplei Twist	95
Ureaplasma parvum serovar 3 ATCC 27815	19087
Ureaplasma urealyticum	1

Our work to assess word usage diversity in proteomes parallels other efforts to describe the potential diversity of protein folds. If amino acid motifs contribute information to a discrete rule set for guiding protein folding, then the finite set of structural folds observed in proteins indicates that amino acid motif utilization is constrained (repetitive) to generate an “ideal form” of a particular protein [37]. Recent efforts to quantify the folding space of proteins suggest the discovery rate of new structural folds is at a plateau [38]. This idea that folding motifs are used over and over again as structural building blocks of proteins implies that the frequencies of amino acid word utilization in a proteome will have some repetitive features related to protein structure/function and lifestyle.

These types of analyses will inform the growing field of synthetic biology [39], [40]. The genetic code alone only scratches the surface of complexity in the biological network of a living cell [41], [42]. Metrics of genome complexity, redundancy, and degeneracy need to be utilized in synthetic biology and in developing new ways to target pathogens. Linkages between a genome and the environment that have shaped its function must be better understood if we are to engineer new genomes to accomplish specific anthropogenic goals with the same efficiency of natural genomes that have been subjected to millions of years of evolutionary selection.

## Materials and Methods

### Data Acquisition and Preliminary Processing

Whole genome sequences were downloaded from the NCBI (www.ncbi.nlm.nih.gov). All genome sequences were clustered at 95% amino acid identity using the program CD-HIT to remove duplicate sequences [43], [44]. Table S1 lists the genomes that were used in this study with additional information regarding their classification as free-living or pathogenic bacteria. A copy of each genome fasta file was randomized by stringing all the AA residues together, then employing a Fisher-Yates shuffling algorithm to randomize the total AA sequence for 10 successive iterations and then re-dividing the total string back into the number and length of the original ORFs. The randomized genome contained the identical number of genes, gene lengths and amino acid usages as the native genome; the only difference was the amino acid order was randomized.

### Amino Acid Usage

A comparison of amino acid frequencies in whole genome sequences between the two groups was performed. A Welsh’s two-sample t-test was used to compare the mean frequencies and test the likelihood that the difference among FREE and PATH observations was not zero. This statistic establishes a 95% confidence interval around the difference means and assigns significance based on how far the observed arithmetic difference is from zero.

### Dictionary Processing

For each dictionary (both native and randomized), amino acids words from 2-to-12 mers were counted and retained if a word were repeated at least twice. We calculated an “expected” count for each word as the average probability of randomly combining the N–1 submers (based on observed frequency of the N–1 submer) with the terminal amino acid residue (based on observed amino acid composition). These are similar methods to those published previously [28], [33]. We calculated the deviation between observed and expected counts within a dictionary as a residual distance (for each word in each genome, the perpendicular distance of the OBS and EXP values from a null selection line of a 1∶1 equilibrium). As an example, these observed counts and residual distances are plotted in Figure 1 for E. coli O157. A genome-wide statistic for summarizing total departure between observed and expected word counts was calculated as a summation of all the individual word residual distances. The residual distance is defined as:
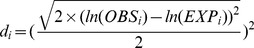
(1)


From this, the summation of all the individual word residual distances for words of length i = 1 to N follows as:
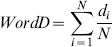
(2)


Repeat counts in Figure 4 were derived from observed counts in the 2-to-12 mer dictionaries. Observed counts were parsed into i bins, where the value in each bin represents the number of unique words repeated i times (e.g., the 10^th^ bin contains the number of words in a dictionary [across all N-mers] that were repeated 10 times). This approach reduced the typical dictionary size from 500,000 words to a 30,000 element vector. More importantly, this vectorization allowed a direct comparison between all genomes, which would be extremely complex with the raw dictionaries. Bin counts were then normalized to the number of total amino acids present in the non-redundant fasta file. The Fmean and Pmean vectors were calculated as the simple mean of each bin position for all FREE and PATH genomes, respectively. The linear discriminant analyses using the normalized repeat count vectors (Figures 4 and 5) were run with two different MDS-LDA approaches: 1) a custom script in MatLab using the “Statistical Pattern Recognition Tools” package (STPRTool; http://cmp.felk.cvut.cz/cmp/software/stprtool/), and 2) the “Multiple Response Permutation Procedure” (MRPP) in the VEGAN package for R Statistics. Both approaches provided nearly identical results. In both MatLab and R, we added an iterative (10 k), Monte Carlo randomization to each script to define the distribution in the random separation between group centroids (Figure 6). To ensure that there were no effects related to chromosome number, pathogenicity islands or plasmids with high concentration of genes from specific functional categories, we repeated the entire analysis on genomes with only one chromosome and no plasmids. The results were similar to Figure 6 and are not shown. This subset contained 482 genomes with 243 free-living and 239 pathogens. The overall variance in word usage data was less variable within this smaller group, and consequently the MDS-LDA analyses revealed differences between the groups that were more statistically significant, although we only report significance here at the p<1e^–06^ level.

## Supporting Information

Table S1 **Free-living and pathogenic bacteria used in analyses.**
(HTML)
