# Supplementary material for: Protein Languages Differ Depending on Microorganism Lifestyle
Source: PLoS One. 2014 May 14;9(5):e96910. doi: 10.1371/journal.pone.0096910 (PMC4020791; doi:10.1371/journal.pone.0096910)
Supplement: Table S1 — Free-living and pathogenic bacteria used in analyses. (HTML) [file pone.0096910.s001.html]

|  |  |  |  |  |  |  |  |  |  |  |  |  |  |  |  |  |  |  |  |  |  |  |
| --- | --- | --- | --- | --- | --- | --- | --- | --- | --- | --- | --- | --- | --- | --- | --- | --- | --- | --- | --- | --- | --- | --- |
| ****Table S1:* Free-living and pathogenic bacteria used in analyses*** | | | | | | | | | | | | | | | | | | | | | | |
| **Genome** | **FreePath** | **King** | **CHR** | **Plasmids** | **Gram** | **O2** | **Habitat** | **Topt** | **Path** | **Disease** |
| Acaryochloris\_marina\_MBIC11017 | **Free** | B | 1 | 9 | NaN | Aerobic | Aquatic | NaN | No | None |
| Acetobacter\_pasteurianus\_IFO\_3283\_01 | **Free** | B | 1 | 6 | - | Aerobic | Multiple | NaN | NaN | NaN |
| Acholeplasma\_laidlawii\_PG\_8A | **Free** | B | 1 | 0 | NaN | Facultative | Specialized | 37 | NaN | NaN |
| Acidimicrobium\_ferrooxidans\_DSM\_10331 | **Free** | B | 1 | 0 | + | Anaerobic | Specialized | NaN | No | NaN |
| Acidiphilium\_cryptum\_JF-5 | **Free** | B | 1 | 8 | - | Aerobic | Multiple | 35 | No | NaN |
| Acidithiobacillus\_ferrooxidans\_ATCC\_53993 | **Free** | B | 1 | 0 | - | Aerobic | Specialized | NaN | No | NaN |
| Candidatus\_Koribacter\_versatilis\_Ellin345 | **Free** | B | 1 | 0 | - | Aerobic | Terrestrial | NaN | No | NaN |
| Acidobacterium\_capsulatum\_ATCC\_51196 | **Free** | B | 1 | 0 | - | Aerobic | Multiple | 30 | No | NaN |
| Acidothermus\_cellulolyticus\_11B | **Free** | B | 1 | 0 | + | Aerobic | Aquatic | 58 | No | NaN |
| Acidovorax\_JS42 | **Free** | B | 1 | 2 | - | Aerobic | Terrestrial | NaN | No | NaN |
| Acidovorax\_avenae\_citrulli\_AAC00-1 | **Path** | B | 1 | 0 | - | Aerobic | Multiple | NaN | Fruit | Bacterial |
| Acinetobacter\_baumannii\_AB307\_0294 | **Path** | B | 1 | 0 | - | Aerobic | Multiple | NaN | Human | Nosocomial |
| Acinetobacter\_sp\_ADP1 | **Free** | B | 1 | 0 | - | Aerobic | Multiple | 37 | Human | Nosocomial |
| Actinobacillus\_pleuropneumoniae\_L20 | **Path** | B | 1 | 0 | - | Facultative | Host-associated | NaN | Porcine | Fibrinous |
| Actinobacillus\_pleuropneumoniae\_serovar\_3\_JL03 | **Path** | B | 1 | 0 | - | Facultative | Host-associated | 37 | Porcine | Fibrinous |
| Actinobacillus\_pleuropneumoniae\_serovar\_7\_AP76 | **Path** | B | 1 | 3 | - | Facultative | Host-associated | NaN | Porcine | Fibrinous |
| Actinosynnema\_mirum\_DSM\_43827 | **Free** | B | 1 | 0 | + | NaN | Terrestrial | NaN | NaN | NaN |
| Aeromonas\_hydrophila\_ATCC\_7966 | **Path** | B | 1 | 0 | - | Facultative | Multiple | NaN | Animal | Gastroenteritis, |
| Aeromonas\_hydrophila\_ATCC\_7966 | **Path** | B | 1 | 0 | - | Facultative | Multiple | NaN | Animal | Gastroenteritis, |
| Aeromonas\_salmonicida\_A449 | **Path** | B | 1 | 5 | - | Facultative | Aquatic | NaN | Fish | Furunculosis |
| Agrobacterium\_radiobacter\_K84 | **Free** | B | 2 | 3 | - | Aerobic | Terrestrial | NaN | No | None |
| Agrobacterium\_vitis\_S4 | **Path** | B | 2 | 5 | - | Aerobic | Multiple | NaN | Grape | Crown |
| Alcanivorax\_borkumensis\_SK2 | **Free** | B | 1 | 0 | - | Aerobic | Aquatic | NaN | No | None |
| Alicyclobacillus\_acidocaldarius\_DSM\_446 | **Free** | B | 1 | 3 | + | Aerobic | Specialized | NaN | No | NaN |
| Aliivibrio\_salmonicida\_LFI1238 | **Path** | B | 2 | 4 | - | Facultative | Aquatic | NaN | Fis | Hitra |
| Aliivibrio\_salmonicida\_LFI1238 | **Path** | B | 2 | 4 | - | Facultative | Aquatic | NaN | Fis | Hitra |
| Alkalilimnicola\_ehrlichei\_MLHE-1 | **Free** | B | 1 | 0 | NaN | Facultative | Aquatic | NaN | No | NaN |
| Alkaliphilus\_oremlandii\_OhILAs | **Free** | B | 1 | 0 | + | Anaerobic | Aquatic | NaN | No | NaN |
| Alteromonas\_macleodii\_\_Deep\_ecotype\_ | **Free** | B | 1 | 0 | - | Aerobic | Aquatic | NaN | No | NaN |
| Ammonifex\_degensii\_KC4 | **Free** | B | 1 | 1 | - | Anaerobic | Specialized | 70 | No | NaN |
| Anabaena\_variabilis\_ATCC\_29413 | **Free** | B | 1 | 3 | NaN | Aerobic | Multiple | NaN | No | NaN |
| Anaerocellum\_thermophilum\_DSM\_6725 | **Free** | B | 1 | 2 | NaN | Anaerobic | Specialized | NaN | No | NaN |
| Anaerococcus\_prevotii\_DSM\_20548 | **Path** | B | 1 | 1 | + | Anaerobic | Host-associated | NaN | Yes | Opportunistic |
| Anaeromyxobacter\_Fw109-5 | **Free** | B | 1 | 0 | NaN | Anaerobic | Terrestrial | NaN | No | None |
| Anaeromyxobacter\_Fw109-5 | **Free** | B | 1 | 0 | NaN | Anaerobic | Terrestrial | NaN | No | None |
| Anaeromyxobacter\_Fw109-5 | **Free** | B | 1 | 0 | NaN | Anaerobic | Terrestrial | NaN | No | None |
| Anaeromyxobacter\_K | **Free** | B | 1 | 0 | NaN | Anaerobic | Terrestrial | NaN | No | None |
| Anaeromyxobacter\_dehalogenans\_2CP-C | **Free** | B | 1 | 0 | - | Facultative | Terrestrial | 30 | No | None |
| Anaeromyxobacter\_dehalogenans\_2CP\_1 | **Free** | B | 1 | 0 | - | Facultative | Terrestrial | NaN | No | None |
| Anaplasma\_centrale\_Israel | **Path** | B | 1 | 0 | NaN | Aerobic | Host-associated | NaN | Cattle | Anaplasmosis |
| Anaplasma\_marginale\_Florida | **Path** | B | 1 | 0 | NaN | Aerobic | Host-associated | NaN | Cattle | Bovine |
| Anoxybacillus\_flavithermus\_WK1 | **Free** | B | 1 | 0 | + | Facultative | Specialized | NaN | No | NaN |
| Arcobacter\_butzleri\_RM4018 | **Path** | B | 1 | 0 | - | Aerobic | Multiple | NaN | Human | Gastroenteritis |
| Aromatoleum\_aromaticum\_EbN1 | **Free** | B | 1 | 2 | - | Facultative | Terrestrial | 26 | No | None |
| Aromatoleum\_aromaticum\_EbN1 | **Free** | B | 1 | 2 | - | Facultative | Terrestrial | 26 | No | None |
| Arthrobacter\_aurescens\_TC1 | **Free** | B | 1 | 2 | + | Aerobic | Terrestrial | 30 | No | None |
| Arthrobacter\_chlorophenolicus\_A6 | **Free** | B | 1 | 2 | + | Aerobic | Terrestrial | NaN | No | NaN |
| Aster\_yellows\_witches-broom\_phytoplasma\_AYWB | **Path** | B | 1 | 4 | NaN | Aerobic | Host-associated | NaN | Plant | Aster |
| Azorhizobium\_caulinodans\_ORS\_571 | **Path** | B | 1 | 0 | NaN | NaN | Host-associated | NaN | NaN | NaN |
| Azorhizobium\_caulinodans\_ORS\_571 | **Path** | B | 1 | 0 | NaN | NaN | Host-associated | NaN | NaN | NaN |
| Azorhizobium\_caulinodans\_ORS\_571 | **Path** | B | 1 | 0 | NaN | NaN | Host-associated | NaN | NaN | NaN |
| Bacillus\_amyloliquefaciens\_FZB42 | **Free** | B | 1 | 0 | + | Aerobic | Terrestrial | NaN | No | NaN |
| Bacillus\_anthracis\_A0248 | **Path** | B | 1 | 2 | + | Facultative | Multiple | NaN | Human, | Anthrax |
| Bacillus\_anthracis\_A0248 | **Path** | B | 1 | 2 | + | Facultative | Multiple | NaN | Human, | Anthrax |
| Bacillus\_anthracis\_Ames\_0581 | **Path** | B | 1 | 2 | + | Facultative | Terrestrial | NaN | Animal | Anthrax |
| Bacillus\_anthracis\_str\_Sterne | **Path** | B | 1 | 0 | + | Facultative | Multiple | NaN | Animal | Anthrax |
| Bacillus\_cereus\_03BB102 | **Path** | B | 1 | 1 | + | Aerobic | Multiple | NaN | Human | Pneumonia |
| Bacillus\_cereus\_AH187 | **Path** | B | 1 | 4 | + | Aerobic | Multiple | NaN | Human | Food |
| Bacillus\_cereus\_AH187 | **Path** | B | 1 | 4 | + | Aerobic | Multiple | NaN | Human | Food |
| Bacillus\_cereus\_AH820 | **Path** | B | 1 | 3 | + | Aerobic | Multiple | NaN | Human | Periodontal |
| Bacillus\_cereus\_B4264 | **Path** | B | 1 | 0 | + | Aerobic | Multiple | 37 | Human | Pneumonia |
| Bacillus\_cereus\_B4264 | **Path** | B | 1 | 0 | + | Aerobic | Multiple | 37 | Human | Pneumonia |
| Bacillus\_cereus\_G9842 | **Path** | B | 1 | 2 | + | Aerobic | Multiple | NaN | Human | Food |
| Bacillus\_cereus\_Q1 | **Free** | B | 1 | 2 | + | Aerobic | Terrestrial | NaN | No | NaN |
| Bacillus\_cereus\_ZK | **Path** | B | 1 | 5 | + | Aerobic | Terrestrial | NaN | Human, | Food |
| Bacillus\_cereus\_cytotoxis\_NVH\_391-98 | **Path** | B | 1 | 1 | + | Aerobic | Terrestrial | NaN | Human | Food |
| Bacillus\_licheniformis\_ATCC\_14580 | **Path** | B | 1 | 0 | + | Facultative | Terrestrial | NaN | Human | Food |
| Bacillus\_licheniformis\_DSM\_13 | **Path** | B | 1 | 0 | + | Facultative | NaN | NaN | Human | Food |
| Bacillus\_pumilus\_SAFR-032 | **Free** | B | 1 | 0 | + | Aerobic | Terrestrial | NaN | No | NaN |
| Bacillus\_thuringiensis\_Al\_Hakam | **Path** | B | 1 | 1 | + | Facultative | Multiple | NaN | Insect | Sotto |
| Bacillus\_thuringiensis\_konkukian | **Path** | B | 1 | 1 | + | Facultative | Multiple | NaN | Insect | Sotto |
| Bacillus\_weihenstephanensis\_KBAB4 | **Path** | B | 1 | 4 | + | Aerobic | Terrestrial | NaN | Human | Food |
| Bacteroides\_fragilis\_YCH46 | **Path** | B | 1 | 1 | - | Anaerobic | NaN | 37 | Human | Severe |
| Bacteroides\_vulgatus\_ATCC\_8482 | **Path** | B | 1 | 0 | + | Anaerobic | Host-associated | NaN | Mammal | Opportunistic |
| Bacteroides\_vulgatus\_ATCC\_8482 | **Path** | B | 1 | 0 | + | Anaerobic | Host-associated | NaN | Mammal | Opportunistic |
| Bartonella\_bacilliformis\_KC583 | **Path** | B | 1 | 0 | - | Aerobic | Host-associated | 28 | Human | Carrion |
| Bartonella\_grahamii\_as4aup | **Path** | B | 1 | 1 | - | Aerobic | Host-associated | NaN | Human, | NaN |
| Bartonella\_tribocorum\_CIP\_105476 | **Free** | B | 1 | 1 | - | Aerobic | Host-associated | NaN | Rat | Bartinellosis |
| Bartonella\_tribocorum\_CIP\_105476 | **Free** | B | 1 | 1 | - | Aerobic | Host-associated | NaN | Rat | Bartinellosis |
| Bartonella\_tribocorum\_CIP\_105476 | **Free** | B | 1 | 1 | - | Aerobic | Host-associated | NaN | Rat | Bartinellosis |
| Bartonella\_tribocorum\_CIP\_105476 | **Free** | B | 1 | 1 | - | Aerobic | Host-associated | NaN | Rat | Bartinellosis |
| Beutenbergia\_cavernae\_DSM\_12333 | **Free** | B | 1 | 0 | + | Aerobic | Terrestrial | NaN | No | NaN |
| Bifidobacterium\_adolescentis\_ATCC\_15703 | **Free** | B | 1 | 0 | + | Anaerobic | Host-associated | 37 | No | None |
| Bifidobacterium\_adolescentis\_ATCC\_15703 | **Free** | B | 1 | 0 | + | Anaerobic | Host-associated | 37 | No | None |
| Bifidobacterium\_adolescentis\_ATCC\_15703 | **Free** | B | 1 | 0 | + | Anaerobic | Host-associated | 37 | No | None |
| Bifidobacterium\_animalis\_lactis\_AD011 | **Free** | B | 1 | 0 | + | Anaerobic | Multiple | NaN | NaN | NaN |
| Bifidobacterium\_animalis\_lactis\_Bl\_04 | **Free** | B | 1 | 0 | + | Anaerobic | Multiple | NaN | NaN | NaN |
| Bifidobacterium\_animalis\_lactis\_DSM\_10140 | **Free** | B | 1 | 0 | + | Anaerobic | Multiple | NaN | No | NaN |
| Bordetella\_petrii | **Free** | B | 1 | 0 | - | Anaerobic | Aquatic | NaN | No | NaN |
| Borrelia\_afzelii\_PKo | **Path** | B | 1 | 8 | - | Aerobic | Host-associated | NaN | Human | Acrodermatitis |
| Borrelia\_burgdorferi\_ZS7 | **Path** | B | 1 | 14 | NaN | Microaerophilic | Host-associated | NaN | Human | Lyme |
| Borrelia\_duttonii\_Ly | **Path** | B | 1 | 16 | NaN | Aerobic | Host-associated | NaN | Human | Tick-borne |
| Borrelia\_garinii\_PBi | **Path** | B | 1 | 3 | NaN | NaN | Host-associated | NaN | Human | Lyme |
| Borrelia\_garinii\_PBi | **Path** | B | 1 | 3 | NaN | NaN | Host-associated | NaN | Human | Lyme |
| Borrelia\_hermsii\_DAH | **Path** | B | 1 | 0 | NaN | Aerobic | Host-associated | NaN | Human | Tick-borne |
| Borrelia\_hermsii\_DAH | **Path** | B | 1 | 0 | NaN | Aerobic | Host-associated | NaN | Human | Tick-borne |
| Borrelia\_recurrentis\_A1 | **Path** | B | 1 | 7 | - | Aerobic | Host-associated | NaN | Human | Louse-borne |
| Borrelia\_turicatae\_91E135 | **Path** | B | 1 | 0 | NaN | Aerobic | Host-associated | NaN | Human | Tick-borne |
| Brachybacterium\_faecium\_DSM\_4810 | **Free** | B | 1 | 0 | + | Aerobic | Terrestrial | NaN | No | None |
| Brachyspira\_hyodysenteriae\_WA1 | **Path** | B | 1 | 1 | - | Facultative | Host-associated | NaN | Yes | Swine |
| Brevibacillus\_brevis\_NBRC\_100599 | **Free** | B | 1 | 0 | + | Aerobic | Terrestrial | NaN | No | NaN |
| Brevibacillus\_brevis\_NBRC\_100599 | **Free** | B | 1 | 0 | + | Aerobic | Terrestrial | NaN | No | NaN |
| Brucella\_abortus\_S19 | **Path** | B | 2 | 0 | - | Facultative | Multiple | 37 | Mammal | Spontaneous |
| Brucella\_canis\_ATCC\_23365 | **Path** | B | 2 | 0 | - | NaN | NaN | NaN | Dog, | Canine |
| Brucella\_canis\_ATCC\_23365 | **Path** | B | 2 | 0 | - | NaN | NaN | NaN | Dog, | Canine |
| Brucella\_canis\_ATCC\_23365 | **Path** | B | 2 | 0 | - | NaN | NaN | NaN | Dog, | Canine |
| Brucella\_canis\_ATCC\_23365 | **Path** | B | 2 | 0 | - | NaN | NaN | NaN | Dog, | Canine |
| Brucella\_melitensis\_ATCC\_23457 | **Path** | B | 2 | 0 | - | Facultative | Host-associated | NaN | Human, | Brucellosis |
| Brucella\_melitensis\_biovar\_Abortus | **Path** | B | 2 | 0 | - | Facultative | Host-associated | NaN | Human, | Brucellosis |
| Brucella\_microti\_CCM\_4915 | **Path** | B | 2 | 0 | NaN | NaN | Multiple | NaN | Mammals | NaN |
| Brucella\_microti\_CCM\_4915 | **Path** | B | 2 | 0 | NaN | NaN | Multiple | NaN | Mammals | NaN |
| Brucella\_microti\_CCM\_4915 | **Path** | B | 2 | 0 | NaN | NaN | Multiple | NaN | Mammals | NaN |
| Brucella\_microti\_CCM\_4915 | **Path** | B | 2 | 0 | NaN | NaN | Multiple | NaN | Mammals | NaN |
| Brucella\_ovis | **Path** | B | 2 | 0 | - | Facultative | Host-associated | 37 | Sheep | Inflammation |
| Brucella\_suis\_ATCC\_23445 | **Path** | B | 2 | 0 | - | Aerobic | Host-associated | 37 | Human, | Brucellosis, |
| Brucella\_suis\_ATCC\_23445 | **Path** | B | 2 | 0 | - | Aerobic | Host-associated | 37 | Human, | Brucellosis, |
| Burkholderia\_383 | **Path** | B | 3 | 0 | - | Facultative | Multiple | NaN | Human | Necrotizing |
| Burkholderia\_ambifaria\_MC40\_6 | **Path** | B | 3 | 1 | - | NaN | Multiple | NaN | Human | Cepacia |
| Burkholderia\_cenocepacia\_AU\_1054 | **Path** | B | 3 | 0 | NaN | NaN | NaN | NaN | Human | Necrotizing |
| Burkholderia\_cenocepacia\_MC0\_3 | **Path** | B | 3 | 0 | - | Facultative | Multiple | NaN | Human | Necrotizing |
| Burkholderia\_cepacia\_AMMD | **Free** | B | 3 | 1 | - | Facultative | Multiple | NaN | NaN | NaN |
| Burkholderia\_glumae\_BGR1 | **Path** | B | 2 | 4 | - | Aerobic | Multiple | NaN | Rice | NaN |
| Burkholderia\_glumae\_BGR1 | **Path** | B | 2 | 4 | - | Aerobic | Multiple | NaN | Rice | NaN |
| Burkholderia\_mallei\_NCTC\_10229 | **Path** | B | 2 | 0 | - | NaN | Host-associated | NaN | Human, | Glanders |
| Burkholderia\_mallei\_NCTC\_10247 | **Path** | B | 2 | 0 | - | NaN | Host-associated | NaN | Human, | Glanders |
| Burkholderia\_mallei\_SAVP1 | **Path** | B | 2 | 0 | - | NaN | Host-associated | NaN | Human, | Glanders |
| Burkholderia\_multivorans\_ATCC\_17616\_JGI | **Path** | B | 3 | 1 | - | Aerobic | Host-associated | 37 | Human | Cepacia |
| Burkholderia\_phytofirmans\_PsJN | **Free** | B | 2 | 1 | - | Aerobic | Terrestrial | 30 | No | NaN |
| Burkholderia\_pseudomallei\_1106a | **Path** | B | 2 | 0 | - | Aerobic | Terrestrial | NaN | Animal | Melioidosis |
| Burkholderia\_pseudomallei\_1710b | **Path** | B | 2 | 0 | - | Aerobic | Terrestrial | NaN | Animal | Melioidosis |
| Burkholderia\_pseudomallei\_668 | **Path** | B | 2 | 0 | - | Aerobic | Terrestrial | NaN | Animal | Melioidosis |
| Burkholderia\_thailandensis\_E264 | **Free** | B | 2 | 0 | - | Aerobic | Terrestrial | NaN | NaN | NaN |
| Burkholderia\_vietnamiensis\_G4 | **Path** | B | 3 | 5 | - | Facultative | Multiple | NaN | Human | Necrotizing |
| Burkholderia\_xenovorans\_LB400 | **Path** | B | 3 | 0 | - | Aerobic | Multiple | 30 | Human, | Opportunistic |
| Caldicellulosiruptor\_saccharolyticus\_DSM\_8903 | **Free** | B | 1 | 0 | + | Anaerobic | Specialized | NaN | No | NaN |
| Campylobacter\_concisus\_13826 | **Path** | B | 1 | 2 | - | Microaerophilic | Host-associated | NaN | Human | Gastroenteritis |
| Campylobacter\_curvus\_525\_92 | **Path** | B | 1 | 0 | - | Microaerophilic | Host-associated | NaN | Human | Gastroenteritis, |
| Campylobacter\_fetus\_82-40 | **Path** | B | 1 | 0 | - | Microaerophilic | Host-associated | NaN | Human, | Infertility, |
| Campylobacter\_jejuni\_81-176 | **Path** | B | 1 | 2 | - | Microaerophilic | Multiple | NaN | Human | Food |
| Campylobacter\_jejuni\_81116 | **Path** | B | 1 | 0 | - | Microaerophilic | Multiple | NaN | Human | Food |
| Campylobacter\_jejuni\_doylei\_269\_97 | **Path** | B | 1 | 0 | - | Microaerophilic | Multiple | NaN | Human | Bacteremia |
| Campylobacter\_jejuni\_doylei\_269\_97 | **Path** | B | 1 | 0 | - | Microaerophilic | Multiple | NaN | Human | Bacteremia |
| Campylobacter\_lari\_RM2100 | **Path** | B | 1 | 1 | - | Microaerophilic | Multiple | NaN | Animal | Gastroenteritis |
| Candidatus\_Accumulibacter\_phosphatis\_clade\_IIA\_UW\_1 | **Free** | B | 1 | 3 | - | NaN | Specialized | NaN | NaN | NaN |
| Candidatus\_Azobacteroides\_pseudotrichonymphae\_genomovar\_\_CFP2 | **Free** | B | 1 | 4 | NaN | NaN | Specialized | NaN | No | NaN |
| Candidatus\_Blochmannia\_floridanus | **Free** | B | 1 | 0 | - | NaN | Specialized | NaN | No | NaN |
| Candidatus\_Carsonella\_ruddii\_PV | **Free** | B | 1 | 0 | NaN | NaN | Specialized | NaN | No | NaN |
| Candidatus\_Desulfococcus\_oleovorans\_Hxd3 | **Free** | B | 1 | 0 | - | Anaerobic | Aquatic | NaN | No | NaN |
| Candidatus\_Desulforudis\_audaxviator\_MP104C | **Free** | B | 1 | 0 | + | NaN | Specialized | NaN | No | NaN |
| Candidatus\_Liberibacter\_asiaticus\_psy62 | **Path** | B | 1 | 0 | NaN | NaN | NaN | NaN | Citrus | Huanglongbing |
| Candidatus\_Methanoregula\_boonei\_6A8 | **Path** | A | 1 | 0 | NaN | Anaerobic | Terrestrial | 37 | No | NaN |
| Candidatus\_Methanoregula\_boonei\_6A8 | **Path** | A | 1 | 0 | NaN | Anaerobic | Terrestrial | 37 | No | NaN |
| Candidatus\_Methanoregula\_boonei\_6A8 | **Path** | A | 1 | 0 | NaN | Anaerobic | Terrestrial | 37 | No | NaN |
| Candidatus\_Pelagibacter\_ubique\_HTCC1062 | **Free** | B | 1 | 0 | - | Aerobic | Aquatic | NaN | NaN | NaN |
| Candidatus\_Pelagibacter\_ubique\_HTCC1062 | **Free** | B | 1 | 0 | - | Aerobic | Aquatic | NaN | NaN | NaN |
| Candidatus\_Pelagibacter\_ubique\_HTCC1062 | **Free** | B | 1 | 0 | - | Aerobic | Aquatic | NaN | NaN | NaN |
| Candidatus\_Phytoplasma\_australiense | **Path** | B | 1 | 0 | NaN | Aerobic | Host-associated | NaN | Grapevines | NaN |
| Candidatus\_Phytoplasma\_mali | **Free** | B | 1 | 0 | NaN | Aerobic | Host-associated | NaN | Plant | Apple |
| Candidatus\_Phytoplasma\_mali | **Free** | B | 1 | 0 | NaN | Aerobic | Host-associated | NaN | Plant | Apple |
| Candidatus\_Sulcia\_muelleri\_GWSS | **Path** | B | 1 | 0 |  |  |  |  |  |  |
| Capnocytophaga\_ochracea\_DSM\_7271 | **Path** | B | 1 | 0 | - | Facultative | Host-associated | NaN | Human | NaN |
| Catenulispora\_acidiphila\_DSM\_44928 | **Free** | B | 1 | 0 | + | Aerobic | Terrestrial | NaN | No | NaN |
| Caulobacter\_K31 | **Path** | B | 1 | 2 | - | Aerobic | NaN | NaN | No | None |
| Caulobacter\_crescentus | **Free** | B | 1 | 0 | - | Aerobic | Aquatic | 35 | No | NaN |
| Caulobacter\_crescentus\_NA1000 | **Free** | B | 1 | 0 | - | Aerobic | Aquatic | NaN | No | NaN |
| Cellvibrio\_japonicus\_Ueda107 | **Free** | B | 1 | 0 | - | Aerobic | Terrestrial | NaN | No | NaN |
| Chitinophaga\_pinensis\_DSM\_2588 | **Free** | B | 1 | 0 | - | Aerobic | Terrestrial | NaN | No | NaN |
| Chlamydia\_trachomatis\_434\_Bu | **Path** | B | 1 | 0 | - | NaN | Host-associated | NaN | Human | Lymphogranuloma |
| Chlamydia\_trachomatis\_A\_HAR-13 | **Path** | B | 1 | 1 | - | NaN | Host-associated | NaN | Human | Pharyngitis, |
| Chlamydia\_trachomatis\_B\_TZ1A828\_OT | **Path** | B | 1 | 0 | - | NaN | Host-associated | NaN | Human | Ocular |
| Chlamydia\_trachomatis\_Jali20 | **Path** | B | 1 | 0 | - | NaN | Host-associated | NaN | Human | NaN |
| Chlamydia\_trachomatis\_Jali20 | **Path** | B | 1 | 0 | - | NaN | Host-associated | NaN | Human | NaN |
| Chlamydia\_trachomatis\_Jali20 | **Path** | B | 1 | 0 | - | NaN | Host-associated | NaN | Human | NaN |
| Chlamydia\_trachomatis\_L2b\_UCH\_1\_proctitis | **Path** | B | 1 | 0 | - | NaN | Host-associated | NaN | Human | Lymphogranuloma |
| Chlamydophila\_felis\_Fe\_C-56 | **Path** | B | 1 | 1 | - | NaN | Host-associated | 37 | Human, | Pharyngitis, |
| Chlamydophila\_pneumoniae\_J138 | **Path** | B | 1 | 0 | - | NaN | Host-associated | 37 | Human | Pharyngitis, |
| Chlamydophila\_pneumoniae\_TW\_183 | **Path** | B | 1 | 0 | - | NaN | Host-associated | 37 | Human | Pharyngitis, |
| Chlamydophila\_pneumoniae\_TW\_183 | **Path** | B | 1 | 0 | - | NaN | Host-associated | 37 | Human | Pharyngitis, |
| Chlorobaculum\_parvum\_NCIB\_8327 | **Free** | B | 1 | 0 | NaN | Facultative | Aquatic | NaN | No | NaN |
| Chlorobium\_chlorochromatii\_CaD3 | **Free** | B | 1 | 0 | - | Anaerobic | Aquatic | NaN | No | NaN |
| Chlorobium\_limicola\_DSM\_245 | **Free** | B | 1 | 0 | - | Anaerobic | Aquatic | NaN | No | None |
| Chlorobium\_phaeobacteroides\_BS1 | **Free** | B | 1 | 0 | - | NaN | Aquatic | NaN | No | None |
| Chlorobium\_phaeobacteroides\_DSM\_266 | **Free** | B | 1 | 0 | - | Facultative | Aquatic | NaN | No | None |
| Chlorobium\_tepidum\_TLS | **Free** | B | 1 | 0 | NaN | Anaerobic | Specialized | 48 | No | NaN |
| Chloroflexus\_aggregans\_DSM\_9485 | **Free** | B | 1 | 0 | NaN | Facultative | Specialized | NaN | No | NaN |
| Chloroherpeton\_thalassium\_ATCC\_35110 | **Free** | B | 1 | 0 | - | Facultative | Aquatic | NaN | No | NaN |
| Chloroherpeton\_thalassium\_ATCC\_35110 | **Free** | B | 1 | 0 | - | Facultative | Aquatic | NaN | No | NaN |
| Citrobacter\_koseri\_ATCC\_BAA-895 | **Path** | B | 1 | 2 | - | NaN | Multiple | NaN | Human, | Bacteremia, |
| Clavibacter\_michiganensis\_NCPPB\_382 | **Path** | B | 1 | 2 | + | Aerobic | Multiple | NaN | Tomato | Tomato |
| Clostridium\_botulinum\_A2\_Kyoto | **Path** | B | 1 | 0 | + | Anaerobic | Multiple | 37 | Human | Botulism |
| Clostridium\_botulinum\_A3\_Loch\_Maree | **Path** | B | 1 | 1 | + | Anaerobic | Multiple | 37 | Human | Botulism |
| Clostridium\_botulinum\_A\_ATCC\_19397 | **Path** | B | 1 | 0 | + | Anaerobic | Multiple | 37 | Human | Botulism |
| Clostridium\_botulinum\_A\_ATCC\_19397 | **Path** | B | 1 | 0 | + | Anaerobic | Multiple | 37 | Human | Botulism |
| Clostridium\_botulinum\_A\_ATCC\_19397 | **Path** | B | 1 | 0 | + | Anaerobic | Multiple | 37 | Human | Botulism |
| Clostridium\_botulinum\_A\_ATCC\_19397 | **Path** | B | 1 | 0 | + | Anaerobic | Multiple | 37 | Human | Botulism |
| Clostridium\_botulinum\_A\_ATCC\_19397 | **Path** | B | 1 | 0 | + | Anaerobic | Multiple | 37 | Human | Botulism |
| Clostridium\_botulinum\_A\_Hall | **Path** | B | 1 | 0 | + | Anaerobic | Multiple | 37 | Human | Botulism |
| Clostridium\_botulinum\_B1\_Okra | **Path** | B | 1 | 1 | + | Anaerobic | Multiple | 37 | Human | Botulism |
| Clostridium\_botulinum\_B1\_Okra | **Path** | B | 1 | 1 | + | Anaerobic | Multiple | 37 | Human | Botulism |
| Clostridium\_botulinum\_B\_Eklund\_17B | **Free** | B | 1 | 1 | + | Anaerobic | Multiple | 37 | No | NaN |
| Clostridium\_botulinum\_B\_Eklund\_17B | **Free** | B | 1 | 1 | + | Anaerobic | Multiple | 37 | No | NaN |
| Clostridium\_botulinum\_Ba4\_657 | **Path** | B | 1 | 2 | NaN | Anaerobic | Multiple | 37 | Human | NaN |
| Clostridium\_botulinum\_E3\_Alaska\_E43 | **Path** | B | 1 | 0 | + | Anaerobic | Multiple | 30 | Human | Botulism |
| Clostridium\_botulinum\_F\_Langeland | **Path** | B | 1 | 1 | + | Anaerobic | Multiple | 37 | Human | Botulism |
| Clostridium\_cellulolyticum\_H10 | **Free** | B | 1 | 0 | + | Anaerobic | Terrestrial | NaN | No | NaN |
| Clostridium\_difficile\_R20291 | **Path** | B | 1 | 0 | + | Anaerobic | Multiple | NaN | Human | Antibiotic-associated |
| Clostridium\_kluyveri\_DSM\_555 | **Free** | B | 1 | 1 | + | Anaerobic | Aquatic | NaN | No | None |
| Clostridium\_kluyveri\_NBRC\_12016 | **Path** | B | 1 | 1 | + | Anaerobic | Aquatic | NaN | No | NaN |
| Clostridium\_kluyveri\_NBRC\_12016 | **Path** | B | 1 | 1 | + | Anaerobic | Aquatic | NaN | No | NaN |
| Clostridium\_kluyveri\_NBRC\_12016 | **Path** | B | 1 | 1 | + | Anaerobic | Aquatic | NaN | No | NaN |
| Clostridium\_novyi\_NT | **Free** | B | 1 | 0 | + | Anaerobic | Terrestrial | NaN | NaN | NaN |
| Clostridium\_perfringens\_SM101 | **Path** | B | 1 | 2 | + | Anaerobic | Multiple | 37 | Human, | Gas |
| Clostridium\_phytofermentans\_ISDg | **Free** | B | 1 | 0 | + | Anaerobic | Terrestrial | 37 | No | NaN |
| Clostridium\_phytofermentans\_ISDg | **Free** | B | 1 | 0 | + | Anaerobic | Terrestrial | 37 | No | NaN |
| Colwellia\_psychrerythraea\_34H | **Free** | B | 1 | 0 | - | Facultative | Specialized | 8 | No | NaN |
| Comamonas\_testosteroni\_CNB\_1 | **Free** | B | 1 | 0 | - | Aerobic | Multiple | NaN | No | NaN |
| Coprothermobacter\_proteolyticus\_DSM\_5265 | **Free** | B | 1 | 0 | - | Anaerobic | Specialized | 63 | No | NaN |
| Corynebacterium\_aurimucosum\_ATCC\_700975 | **Path** | B | 1 | 1 | + | Facultative | Host-associated | NaN | Human | Opportunistic |
| Corynebacterium\_efficiens\_YS-314 | **Free** | B | 1 | 2 | + | Facultative | Multiple | NaN | No | None |
| Corynebacterium\_glutamicum\_ATCC\_13032\_Bielefeld | **Free** | B | 1 | 0 | + | Facultative | Multiple | NaN | No | None |
| Corynebacterium\_glutamicum\_ATCC\_13032\_Kitasato | **Free** | B | 1 | 0 | + | Facultative | Multiple | NaN | No | None |
| Corynebacterium\_glutamicum\_R | **Free** | B | 1 | 1 | + | Facultative | Multiple | NaN | NaN | NaN |
| Corynebacterium\_urealyticum\_DSM\_7109 | **Path** | B | 1 | 0 | + | Aerobic | Host-associated | NaN | Human | Urinary |
| Coxiella\_burnetii\_CbuG\_Q212 | **Path** | B | 1 | 0 | - | Facultative | Multiple | NaN | Animal, | Q |
| Coxiella\_burnetii\_CbuG\_Q212 | **Path** | B | 1 | 0 | - | Facultative | Multiple | NaN | Animal, | Q |
| Coxiella\_burnetii\_CbuK\_Q154 | **Path** | B | 1 | 1 | - | Facultative | Multiple | NaN | Animal, | Q |
| Coxiella\_burnetii\_Dugway\_7E9-12 | **Path** | B | 1 | 1 | - | Facultative | Host-associated | 37 | Animal, | Q-fever |
| Coxiella\_burnetii\_RSA\_331 | **Path** | B | 1 | 1 | - | Facultative | Multiple | NaN | Animal, | Q |
| Cryptobacterium\_curtum\_DSM\_15641 | **Path** | B | 1 | 0 | + | Anaerobic | NaN | NaN | Human | Caries |
| Cyanobacteria\_bacterium\_Yellowstone\_A-Prime | **Free** | B | 1 | 0 | - | Facultative | Specialized | NaN | No | NaN |
| Cyanobacteria\_bacterium\_Yellowstone\_B-Prime | **Free** | B | 1 | 0 | - | Facultative | Specialized | NaN | No | NaN |
| Cyanothece\_ATCC\_51142 | **Free** | B | 2 | 4 | NaN | Facultative | Aquatic | NaN | No | NaN |
| Cyanothece\_PCC\_7424 | **Free** | B | 1 | 6 | NaN | Facultative | Aquatic | NaN | No | NaN |
| Cyanothece\_PCC\_7424 | **Free** | B | 1 | 6 | NaN | Facultative | Aquatic | NaN | No | NaN |
| Cyanothece\_PCC\_7425 | **Free** | B | 1 | 3 | NaN | Facultative | Aquatic | NaN | No | NaN |
| Cyanothece\_PCC\_8801 | **Free** | B | 1 | 3 | NaN | Facultative | Aquatic | NaN | No | NaN |
| Cyanothece\_PCC\_8802 | **Free** | B | 1 | 4 | NaN | Facultative | Aquatic | NaN | No | NaN |
| Cytophaga\_hutchinsonii\_ATCC\_33406 | **Path** | B | 1 | 0 | - | Aerobic | Multiple | 30 | No | NaN |
| Dechloromonas\_aromatica\_RCB | **Free** | B | 1 | 0 | - | Facultative | Multiple | NaN | No | NaN |
| Dehalococcoides\_CBDB1 | **Free** | B | 1 | 0 | + | Anaerobic | Multiple | NaN | No | NaN |
| Dehalococcoides\_VS | **Free** | B | 1 | 0 | NaN | Anaerobic | Aquatic | NaN | No | None |
| Dehalococcoides\_ethenogenes\_195 | **Free** | B | 1 | 0 | + | Anaerobic | Multiple | 35 | No | None |
| Deinococcus\_deserti\_VCD115 | **Free** | B | 1 | 3 | - | Aerobic | Terrestrial | NaN | NaN | NaN |
| Deinococcus\_geothermalis\_DSM\_11300 | **Free** | B | 1 | 2 | + | Aerobic | Aquatic | 47 | No | NaN |
| Delftia\_acidovorans\_SPH-1 | **Free** | B | 1 | 0 | - | Aerobic | Multiple | 30 | NaN | NaN |
| Desulfatibacillum\_alkenivorans\_AK\_01 | **Free** | B | 1 | 0 | - | Anaerobic | Aquatic | NaN | No | NaN |
| Desulfitobacterium\_hafniense\_Y51 | **Free** | B | 1 | 0 | - | Anaerobic | Specialized | NaN | NaN | NaN |
| Desulfobacterium\_autotrophicum\_HRM2 | **Free** | B | 1 | 1 | - | Anaerobic | Multiple | NaN | No | NaN |
| Desulfohalobium\_retbaense\_DSM\_5692 | **Free** | B | 1 | 1 | - | Anaerobic | Specialized | 37 | No | NaN |
| Desulfomicrobium\_baculatum\_DSM\_4028 | **Free** | B | 1 | 0 | - | Anaerobic | Terrestrial | NaN | No | NaN |
| Desulfomicrobium\_baculatum\_DSM\_4028 | **Free** | B | 1 | 0 | - | Anaerobic | Terrestrial | NaN | No | NaN |
| Desulfotalea\_psychrophila\_LSv54 | **Free** | B | 1 | 2 | - | Anaerobic | Specialized | 7 | No | NaN |
| Desulfotomaculum\_acetoxidans\_DSM\_771 | **Free** | B | 1 | 0 | + | Anaerobic | Multiple | NaN | No | NaN |
| Desulfotomaculum\_reducens\_MI-1 | **Free** | B | 1 | 0 | + | Anaerobic | Aquatic | 37 | No | NaN |
| Desulfovibrio\_desulfuricans\_ATCC\_27774 | **Free** | B | 1 | 0 | - | Anaerobic | Multiple | NaN | No | NaN |
| Desulfovibrio\_salexigens\_DSM\_2638 | **Path** | B | 1 | 0 | - | Anaerobic | Multiple | NaN | No | NaN |
| Desulfovibrio\_salexigens\_DSM\_2638 | **Path** | B | 1 | 0 | - | Anaerobic | Multiple | NaN | No | NaN |
| Desulfovibrio\_vulgaris\_DP4 | **Free** | B | 1 | 1 | - | Anaerobic | Multiple | NaN | No | None |
| Desulfovibrio\_vulgaris\_\_Miyazaki\_F\_ | **Path** | B | 1 | 0 | - | Anaerobic | Multiple | NaN | No | NaN |
| Desulfovibrio\_vulgaris\_\_Miyazaki\_F\_ | **Path** | B | 1 | 0 | - | Anaerobic | Multiple | NaN | No | NaN |
| Diaphorobacter\_TPSY | **Free** | B | 1 | 0 | - | Facultative | Aquatic | NaN | No | NaN |
| Dickeya\_dadantii\_Ech586 | **Path** | B | 1 | 0 | - | Facultative | Multiple | NaN | Plant | Soft |
| Dickeya\_dadantii\_Ech703 | **Path** | B | 1 | 0 | - | Facultative | Multiple | NaN | Plant | Soft |
| Dickeya\_dadantii\_Ech703 | **Path** | B | 1 | 0 | - | Facultative | Multiple | NaN | Plant | Soft |
| Dictyoglomus\_thermophilum\_H\_6\_12 | **Free** | B | 1 | 0 | - | Anaerobic | Aquatic | 78 | No | NaN |
| Dictyoglomus\_turgidum\_DSM\_6724 | **Path** | B | 1 | 0 | + | Anaerobic | Specialized | NaN | No | NaN |
| Dictyoglomus\_turgidum\_DSM\_6724 | **Path** | B | 1 | 0 | + | Anaerobic | Specialized | NaN | No | NaN |
| Dinoroseobacter\_shibae\_DFL\_12 | **Path** | B | 1 | 5 | - | Aerobic | NaN | 33 | NaN | NaN |
| Edwardsiella\_ictaluri\_93\_146 | **Path** | B | 1 | 0 | - | Facultative | Aquatic | NaN | Catfish | Enteric |
| Edwardsiella\_tarda\_EIB202 | **Path** | B | 1 | 1 | - | Facultative | Multiple | NaN | Fish, | Systemic |
| Eggerthella\_lenta\_DSM\_2243 | **Path** | B | 1 | 0 | + | Anaerobic | Host-associated | NaN | Human | rare |
| Ehrlichia\_canis\_Jake | **Path** | B | 1 | 0 | - | NaN | Host-associated | NaN | Dog | Ehrlichiosis |
| Ehrlichia\_ruminantium\_Gardel | **Path** | B | 1 | 0 | - | NaN | Host-associated | NaN | Ruminant | African |
| Ehrlichia\_ruminantium\_Welgevonden\_UPSA | **Path** | B | 1 | 0 | - | NaN | Host-associated | NaN | Ruminant | Heartwater |
| Ehrlichia\_ruminantium\_str.\_Welgevonden\_CIRAD | **Path** | B | 1 | 0 | - | NaN | Host-associated | NaN | Ruminant | Heartwater |
| Enterobacter\_sakazakii\_ATCC\_BAA-894 | **Path** | B | 1 | 2 | - | Anaerobic | Host-associated | 37 | Human | Meningitis, |
| Erwinia\_carotovora\_atroseptica\_SCRI1043 | **Path** | B | 1 | 0 | - | Facultative | Multiple | NaN | Plant | Soft |
| Erythrobacter\_litoralis\_HTCC2594 | **Path** | B | 1 | 0 | - | Aerobic | Aquatic | NaN | No | None |
| Erythrobacter\_litoralis\_HTCC2594 | **Path** | B | 1 | 0 | - | Aerobic | Aquatic | NaN | No | None |
| Erythrobacter\_litoralis\_HTCC2594 | **Path** | B | 1 | 0 | - | Aerobic | Aquatic | NaN | No | None |
| Erythrobacter\_litoralis\_HTCC2594 | **Path** | B | 1 | 0 | - | Aerobic | Aquatic | NaN | No | None |
| Escherichia\_coli\_0127\_H6\_E2348\_69 | **Path** | B | 1 | 2 | - | Facultative | Host-associated | 37 | Human | NaN |
| Escherichia\_coli\_0127\_H6\_E2348\_69 | **Path** | B | 1 | 2 | - | Facultative | Host-associated | 37 | Human | NaN |
| Escherichia\_coli\_536 | **Path** | B | 1 | 0 | - | Facultative | Host-associated | NaN | Human | Urinary |
| Escherichia\_coli\_55989 | **Path** | B | 1 | 0 | - | Facultative | Multiple | NaN | Human | Gastroenteritis |
| Escherichia\_coli\_APEC\_O1 | **Path** | B | 1 | 2 | - | Facultative | Host-associated | NaN | Avian | Colibacillosis |
| Escherichia\_coli\_BL21\_DE3\_ | **Free** | B | 1 | 0 | NaN | Anaerobic | Multiple | NaN | NaN | NaN |
| Escherichia\_coli\_BW2952 | **Free** | B | 1 | 0 | - | Facultative | Multiple | NaN | NaN | NaN |
| Escherichia\_coli\_B\_REL606 | **Free** | B | 1 | 0 | - | Facultative | Multiple | NaN | NaN | NaN |
| Escherichia\_coli\_E24377A | **Path** | B | 1 | 6 | - | Facultative | Host-associated | 37 | Human | Diarrhea |
| Escherichia\_coli\_ED1a | **Path** | B | 1 | 0 | - | Facultative | Multiple | NaN | Human | Gastroenteritis |
| Escherichia\_coli\_ED1a | **Path** | B | 1 | 0 | - | Facultative | Multiple | NaN | Human | Gastroenteritis |
| Escherichia\_coli\_IAI1 | **Free** | B | 1 | 0 | - | Facultative | Multiple | NaN | NaN | NaN |
| Escherichia\_coli\_IAI39 | **Path** | B | 1 | 0 | - | Facultative | Multiple | NaN | Human | Urinary |
| Escherichia\_coli\_K\_12\_substr\_\_DH10B | **Free** | B | 1 | 0 | - | Facultative | Host-associated | 37 | No | NaN |
| Escherichia\_coli\_K\_12\_substr\_\_MG1655 | **Free** | B | 1 | 0 | - | Facultative | Host-associated | 37 | No | NaN |
| Escherichia\_coli\_O157H7 | **Path** | B | 1 | 2 | - | Facultative | Host-associated | 37 | Human | Hemorrhagic |
| Escherichia\_coli\_O157\_H7\_EC4115 | **Path** | B | 1 | 2 | - | Facultative | Multiple | 37 | Human | Hemorrhagic |
| Escherichia\_coli\_O157\_H7\_TW14359 | **Path** | B | 1 | 1 | - | Facultative | Multiple | NaN | Human | Hemorrhagic |
| Escherichia\_coli\_O26\_H11\_11368 | **Path** | B | 1 | 3 | - | Facultative | Multiple | 37 | Human | Hemorrhagic |
| Escherichia\_coli\_S88 | **Path** | B | 1 | 1 | - | Facultative | Multiple | NaN | Human | Gastroenteritis, |
| Escherichia\_coli\_SMS\_3\_5 | **Free** | B | 1 | 4 | - | Facultative | Multiple | 37 | No | None |
| Escherichia\_coli\_UMN026 | **Path** | B | 1 | 2 | - | Facultative | Multiple | NaN | Human | Urinary |
| Escherichia\_coli\_UTI89 | **Path** | B | 1 | 1 | - | Facultative | Host-associated | NaN | Human | Cystitis |
| Escherichia\_fergusonii\_ATCC\_35469 | **Path** | B | 1 | 1 | - | Facultative | Multiple | NaN | Human, | Wound |
| Exiguobacterium\_AT1b | **Free** | B | 1 | 0 | + | NaN | Specialized | NaN | No | NaN |
| Exiguobacterium\_sibiricum\_255\_15 | **Free** | B | 1 | 2 | + | Facultative | Specialized | NaN | NaN | NaN |
| Fervidobacterium\_nodosum\_Rt17-B1 | **Free** | B | 1 | 0 | - | Anaerobic | Specialized | 70 | No | NaN |
| Finegoldia\_magna\_ATCC\_29328 | **Path** | B | 1 | 1 | + | Anaerobic | Multiple | NaN | Human | NaN |
| Flavobacterium\_johnsoniae\_UW101 | **Free** | B | 1 | 0 | - | Aerobic | Multiple | NaN | No | NaN |
| Flavobacterium\_psychrophilum\_JIP02\_86 | **Path** | B | 1 | 0 | - | Aerobic | Aquatic | NaN | Salmonid | Cold |
| Francisella\_philomiragia\_ATCC\_25017 | **Path** | B | 1 | 1 | - | Aerobic | Multiple | NaN | Human, | Bacteremia, |
| Francisella\_tularensis\_FSC\_198 | **Path** | B | 1 | 0 | - | Aerobic | Aquatic | NaN | Human, | Tularemia |
| Francisella\_tularensis\_WY96-3418 | **Path** | B | 1 | 0 | - | Aerobic | Host-associated | NaN | Mammal | Tularemia |
| Francisella\_tularensis\_holarctica | **Path** | B | 1 | 0 | - | Aerobic | Host-associated | NaN | Human, | Tularemia |
| Francisella\_tularensis\_holarctica\_FTNF002\_00 | **Path** | B | 1 | 0 | - | Aerobic | Multiple | NaN | Human, | Tularemia |
| Francisella\_tularensis\_holarctica\_OSU18 | **Path** | B | 1 | 0 | - | Aerobic | Multiple | NaN | Human, | Tularemia |
| Francisella\_tularensis\_mediasiatica\_FSC147 | **Path** | B | 1 | 0 | - | Aerobic | Host-associated | NaN | Human, | Tularemia |
| Francisella\_tularensis\_novicida\_U112 | **Path** | B | 1 | 0 | - | Aerobic | Multiple | NaN | NaN | NaN |
| Francisella\_tularensis\_novicida\_U112 | **Path** | B | 1 | 0 | - | Aerobic | Multiple | NaN | NaN | NaN |
| Francisella\_tularensis\_novicida\_U112 | **Path** | B | 1 | 0 | - | Aerobic | Multiple | NaN | NaN | NaN |
| Frankia\_CcI3 | **Free** | B | 1 | 0 | + | Aerobic | Multiple | NaN | NaN | NaN |
| Frankia\_EAN1pec | **Free** | B | 1 | 0 | + | Aerobic | Multiple | NaN | NaN | NaN |
| Fusobacterium\_nucleatum | **Path** | B | 1 | 0 | - | Anaerobic | Host-associated | 37 | Human, | Periodontal |
| Geobacillus\_Y412MC61 | **Free** | B | 1 | 1 | + | Facultative | Specialized | NaN | No | NaN |
| Geobacillus\_kaustophilus\_HTA426 | **Free** | B | 1 | 1 | + | Aerobic | Aquatic | NaN | NaN | NaN |
| Geobacillus\_thermodenitrificans\_NG80-2 | **Free** | B | 1 | 1 | + | Facultative | Specialized | 65 | NaN | NaN |
| Geobacillus\_thermodenitrificans\_NG80-2 | **Free** | B | 1 | 1 | + | Facultative | Specialized | 65 | NaN | NaN |
| Geobacter\_FRC\_32 | **Free** | B | 1 | 0 | - | Anaerobic | Multiple | NaN | No | NaN |
| Geobacter\_M21 | **Free** | B | 1 | 0 | - | Anaerobic | Aquatic | NaN | NaN | NaN |
| Geobacter\_bemidjiensis\_Bem | **Free** | B | 1 | 0 | - | Anaerobic | Terrestrial | 30 | No | NaN |
| Geobacter\_lovleyi\_SZ | **Free** | B | 1 | 1 | - | Anaerobic | Multiple | 35 | No | None |
| Geobacter\_uraniumreducens\_Rf4 | **Free** | B | 1 | 0 | - | Microaerophilic | Multiple | NaN | NaN | NaN |
| Gloeobacter\_violaceus | **Free** | B | 1 | 0 | NaN | NaN | Terrestrial | NaN | NaN | NaN |
| Gluconobacter\_oxydans\_621H | **Free** | B | 1 | 5 | - | Aerobic | Multiple | NaN | NaN | NaN |
| Gordonia\_bronchialis\_DSM\_43247 | **Free** | B | 1 | 1 | + | Aerobic | Host-associated | NaN | Human | Septicemia, |
| Gordonia\_bronchialis\_DSM\_43247 | **Free** | B | 1 | 1 | + | Aerobic | Host-associated | NaN | Human | Septicemia, |
| Gordonia\_bronchialis\_DSM\_43247 | **Free** | B | 1 | 1 | + | Aerobic | Host-associated | NaN | Human | Septicemia, |
| Gordonia\_bronchialis\_DSM\_43247 | **Free** | B | 1 | 1 | + | Aerobic | Host-associated | NaN | Human | Septicemia, |
| Gordonia\_bronchialis\_DSM\_43247 | **Free** | B | 1 | 1 | + | Aerobic | Host-associated | NaN | Human | Septicemia, |
| Gordonia\_bronchialis\_DSM\_43247 | **Free** | B | 1 | 1 | + | Aerobic | Host-associated | NaN | Human | Septicemia, |
| Gramella\_forsetii\_KT0803 | **Free** | B | 1 | 0 | - | Aerobic | Multiple | NaN | NaN | NaN |
| Gramella\_forsetii\_KT0803 | **Free** | B | 1 | 0 | - | Aerobic | Multiple | NaN | NaN | NaN |
| Gramella\_forsetii\_KT0803 | **Free** | B | 1 | 0 | - | Aerobic | Multiple | NaN | NaN | NaN |
| Granulobacter\_bethesdensis\_CGDNIH1 | **Free** | B | 1 | 0 | - | NaN | Multiple | NaN | Human | Chronic |
| Granulobacter\_bethesdensis\_CGDNIH1 | **Free** | B | 1 | 0 | - | NaN | Multiple | NaN | Human | Chronic |
| Haemophilus\_influenzae\_86\_028NP | **Path** | B | 1 | 0 | - | Facultative | Host-associated | NaN | Human | Meningitis, |
| Haemophilus\_influenzae\_PittEE | **Path** | B | 1 | 0 | - | Facultative | Host-associated | NaN | Human | Meningitis, |
| Haemophilus\_influenzae\_PittGG | **Free** | B | 1 | 0 | - | Facultative | Host-associated | NaN | Human | Meningitis, |
| Haemophilus\_influenzae\_PittGG | **Free** | B | 1 | 0 | - | Facultative | Host-associated | NaN | Human | Meningitis, |
| Haemophilus\_parasuis\_SH0165 | **Path** | B | 1 | 0 | - | Facultative | Host-associated | NaN | Porcine | Glasser |
| Hahella\_chejuensis\_KCTC\_2396 | **Free** | B | 1 | 0 | - | Facultative | Aquatic | NaN | No | NaN |
| Haliangium\_ochraceum\_DSM\_14365 | **Free** | B | 1 | 0 | - | Aerobic | Aquatic | NaN | No | NaN |
| Halomicrobium\_mukohataei\_DSM\_12286 | **Path** | A | 1 | 1 | NaN | Facultative | Specialized | 45 | No | NaN |
| Halothermothrix\_orenii\_H\_168 | **Free** | B | 1 | 0 | - | Anaerobic | Aquatic | 60 | No | NaN |
| Halothiobacillus\_neapolitanus\_c2 | **Free** | B | 1 | 0 | - | Aerobic | Specialized | NaN | No | NaN |
| Helicobacter\_acinonychis\_Sheeba | **Path** | B | 1 | 1 | - | Microaerophilic | Host-associated | 37 | Feline | Gastric |
| Helicobacter\_acinonychis\_Sheeba | **Path** | B | 1 | 1 | - | Microaerophilic | Host-associated | 37 | Feline | Gastric |
| Helicobacter\_acinonychis\_Sheeba | **Path** | B | 1 | 1 | - | Microaerophilic | Host-associated | 37 | Feline | Gastric |
| Helicobacter\_acinonychis\_Sheeba | **Path** | B | 1 | 1 | - | Microaerophilic | Host-associated | 37 | Feline | Gastric |
| Helicobacter\_acinonychis\_Sheeba | **Path** | B | 1 | 1 | - | Microaerophilic | Host-associated | 37 | Feline | Gastric |
| Helicobacter\_pylori\_26695 | **Path** | B | 1 | 0 | - | Aerobic | Host-associated | 37 | Human | Gastric |
| Helicobacter\_pylori\_26695 | **Path** | B | 1 | 0 | - | Aerobic | Host-associated | 37 | Human | Gastric |
| Helicobacter\_pylori\_26695 | **Path** | B | 1 | 0 | - | Aerobic | Host-associated | 37 | Human | Gastric |
| Helicobacter\_pylori\_B38 | **Path** | B | 1 | 0 | - | Aerobic | Host-associated | NaN | Human | Gastric |
| Helicobacter\_pylori\_HPAG1 | **Path** | B | 1 | 1 | - | Aerobic | Host-associated | 37 | Human | Gastric |
| Helicobacter\_pylori\_P12 | **Path** | B | 1 | 1 | - | Microaerophilic | Host-associated | NaN | Human | Gastric |
| Helicobacter\_pylori\_P12 | **Path** | B | 1 | 1 | - | Microaerophilic | Host-associated | NaN | Human | Gastric |
| Helicobacter\_pylori\_Shi470 | **Path** | B | 1 | 0 | - | Aerobic | Host-associated | NaN | Human | Gastric |
| Heliobacterium\_modesticaldum\_Ice1 | **Free** | B | 1 | 0 | - | Anaerobic | Multiple | NaN | NaN | NaN |
| Herminiimonas\_arsenicoxydans | **Free** | B | 1 | 0 | NaN | Anaerobic | Aquatic | NaN | No | NaN |
| Herpetosiphon\_aurantiacus\_ATCC\_23779 | **Free** | B | 1 | 2 | - | Aerobic | Multiple | NaN | NaN | NaN |
| Hirschia\_baltica\_ATCC\_49814 | **Free** | B | 1 | 1 | NaN | Aerobic | Aquatic | NaN | No | NaN |
| Hydrogenobaculum\_Y04AAS1 | **Free** | B | 1 | 0 | - | Aerobic | Aquatic | 58 | No | NaN |
| Hyphomonas\_neptunium\_ATCC\_15444 | **Path** | B | 1 | 0 | - | Aerobic | Aquatic | 37 | No | NaN |
| Hyphomonas\_neptunium\_ATCC\_15444 | **Path** | B | 1 | 0 | - | Aerobic | Aquatic | 37 | No | NaN |
| Idiomarina\_loihiensis\_L2TR | **Free** | B | 1 | 0 | - | Aerobic | Specialized | NaN | NaN | NaN |
| Jannaschia\_CCS1 | **Free** | B | 1 | 1 | - | Aerobic | Aquatic | 30 | No | NaN |
| Janthinobacterium\_Marseille | **Free** | B | 1 | 0 | NaN | NaN | Aquatic | NaN | NaN | NaN |
| Kangiella\_koreensis\_DSM\_16069 | **Free** | B | 1 | 0 | - | Facultative | Aquatic | NaN | No | NaN |
| Kineococcus\_radiotolerans\_SRS30216 | **Free** | B | 1 | 2 | + | Aerobic | Multiple | 32 | No | None |
| Kineococcus\_radiotolerans\_SRS30216 | **Free** | B | 1 | 2 | + | Aerobic | Multiple | 32 | No | None |
| Klebsiella\_pneumoniae\_342 | **Free** | B | 1 | 2 | - | Facultative | Host-associated | NaN | No | NaN |
| Klebsiella\_pneumoniae\_NTUH\_K2044 | **Path** | B | 1 | 0 | - | Facultative | Multiple | NaN | Human | Pneumonia |
| Kocuria\_rhizophila\_DC2201 | **Free** | B | 1 | 0 | + | Aerobic | Multiple | NaN | No | NaN |
| Kosmotoga\_olearia\_TBF\_19\_5\_1 | **Free** | B | 1 | 0 | - | NaN | Aquatic | NaN | No | NaN |
| Kytococcus\_sedentarius\_DSM\_20547 | **Free** | B | 1 | 0 | + | Aerobic | NaN | NaN | NaN | NaN |
| Lactobacillus\_casei | **Free** | B | 1 | 0 | + | Facultative | Specialized | NaN | No | NaN |
| Lactobacillus\_casei | **Free** | B | 1 | 0 | + | Facultative | Specialized | NaN | No | NaN |
| Lactobacillus\_casei | **Free** | B | 1 | 0 | + | Facultative | Specialized | NaN | No | NaN |
| Lactobacillus\_casei | **Free** | B | 1 | 0 | + | Facultative | Specialized | NaN | No | NaN |
| Lactobacillus\_delbrueckii\_bulgaricus | **Free** | B | 1 | 0 | + | Facultative | Multiple | NaN | No | NaN |
| Lactobacillus\_fermentum\_IFO\_3956 | **Free** | B | 1 | 0 | + | Facultative | Multiple | NaN | NaN | NaN |
| Lactobacillus\_gasseri\_ATCC\_33323 | **Path** | B | 1 | 0 | + | Facultative | Host-associated | NaN | No | NaN |
| Lactobacillus\_gasseri\_ATCC\_33323 | **Path** | B | 1 | 0 | + | Facultative | Host-associated | NaN | No | NaN |
| Lactobacillus\_gasseri\_ATCC\_33323 | **Path** | B | 1 | 0 | + | Facultative | Host-associated | NaN | No | NaN |
| Lactobacillus\_helveticus\_DPC\_4571 | **Free** | B | 1 | 0 | + | Facultative | Multiple | NaN | No | None |
| Lactobacillus\_helveticus\_DPC\_4571 | **Free** | B | 1 | 0 | + | Facultative | Multiple | NaN | No | None |
| Lactobacillus\_plantarum\_JDM1 | **Free** | B | 1 | 0 |  |  |  |  |  |  |
| Lactobacillus\_plantarum\_JDM1 | **Free** | B | 1 | 0 |  |  |  |  |  |  |
| Lactobacillus\_reuteri\_DSM\_20016 | **Free** | B | 1 | 0 | + | Facultative | Multiple | NaN | No | NaN |
| Lactobacillus\_rhamnosus\_GG | **Path** | B | 1 | 0 | + | Facultative | Multiple | NaN | No | NaN |
| Lactobacillus\_rhamnosus\_GG | **Path** | B | 1 | 0 | + | Facultative | Multiple | NaN | No | NaN |
| Lactobacillus\_rhamnosus\_Lc\_705 | **Free** | B | 1 | 1 | + | Facultative | Multiple | NaN | No | NaN |
| Lactobacillus\_sakei\_23K | **Free** | B | 1 | 0 | + | Facultative | Multiple | NaN | NaN | NaN |
| Lactococcus\_lactis\_cremoris\_MG1363 | **Free** | B | 1 | 0 | + | Facultative | Multiple | 40 | No | None |
| Laribacter\_hongkongensis\_HLHK9 | **Free** | B | 1 | 0 | - | Anaerobic | Host-associated | NaN | Human | Gastroenteritis, |
| Laribacter\_hongkongensis\_HLHK9 | **Free** | B | 1 | 0 | - | Anaerobic | Host-associated | NaN | Human | Gastroenteritis, |
| Legionella\_pneumophila\_Corby | **Path** | B | 1 | 0 | - | Aerobic | Host-associated | NaN | Human | Legionnaire |
| Legionella\_pneumophila\_Lens | **Path** | B | 1 | 1 | - | Aerobic | Host-associated | NaN | Animal | Legionnaire |
| Legionella\_pneumophila\_Paris | **Path** | B | 1 | 1 | - | Aerobic | Host-associated | NaN | Animal | Legionnaire |
| Leptospira\_biflexa\_serovar\_Patoc\_\_Patoc\_1\_\_Ames\_ | **Free** | B | 2 | 1 | - | Aerobic | Multiple | NaN | NaN | NaN |
| Leptospira\_biflexa\_serovar\_Patoc\_\_Patoc\_1\_\_Paris\_ | **Free** | B | 2 | 1 | - | Aerobic | Multiple | NaN | NaN | NaN |
| Leptospira\_borgpetersenii\_serovar\_Hardjo-bovis\_JB197 | **Path** | B | 2 | 0 | - | Aerobic | Host-associated | NaN | Cattle, | Leptospirosis |
| Leptospira\_borgpetersenii\_serovar\_Hardjo-bovis\_L550 | **Path** | B | 2 | 0 | - | Aerobic | Host-associated | NaN | Cattle, | Leptospirosis |
| Leptospira\_interrogans\_serovar\_Copenhageni | **Path** | B | 2 | 0 | NaN | Aerobic | Host-associated | NaN | Animal | Leptospirosis |
| Leptospira\_interrogans\_serovar\_Lai | **Path** | B | 2 | 0 | NaN | Aerobic | Host-associated | NaN | Animal | Leptospirosis |
| Leptothrix\_cholodnii\_SP\_6 | **Free** | B | 1 | 0 | NaN | Aerobic | Aquatic | NaN | No | NaN |
| Leptotrichia\_buccalis\_DSM\_1135 | **Path** | B | 1 | 0 | - | Anaerobic | Host-associated | NaN | Human | Rare |
| Listeria\_monocytogenes | **Path** | B | 1 | 0 | + | Facultative | Multiple | NaN | Human | Listeriosis |
| Listeria\_monocytogenes\_Clip81459 | **Free** | B | 1 | 0 |  |  |  |  |  |  |
| Listeria\_monocytogenes\_HCC23 | **Path** | B | 1 | 0 | + | Facultative | Host-associated | NaN | Human | Listeriosis |
| Listeria\_welshimeri\_serovar\_6b\_SLCC5334 | **Free** | B | 1 | 0 | + | Facultative | Multiple | NaN | No | NaN |
| Lysinibacillus\_sphaericus\_C3\_41 | **Path** | B | 1 | 1 | + | Aerobic | Specialized | NaN | Mosquito | Larvicidal |
| Lysinibacillus\_sphaericus\_C3\_41 | **Path** | B | 1 | 1 | + | Aerobic | Specialized | NaN | Mosquito | Larvicidal |
| Macrococcus\_caseolyticus\_JCSC5402 | **Free** | B | 1 | 8 | + | Facultative | Multiple | 35 | No | None |
| Magnetococcus\_MC-1 | **Free** | B | 1 | 0 | - | Facultative | Aquatic | NaN | No | NaN |
| Magnetospirillum\_magneticum\_AMB-1 | **Free** | B | 1 | 0 | - | Microaerophilic | Aquatic | NaN | No | NaN |
| Maricaulis\_maris\_MCS10 | **Free** | B | 1 | 0 | - | Facultative | Aquatic | NaN | No | NaN |
| Marinobacter\_aquaeolei\_VT8 | **Free** | B | 1 | 2 | - | Facultative | Aquatic | 30 | No | NaN |
| Marinomonas\_MWYL1 | **Free** | B | 1 | 0 | - | Aerobic | Aquatic | NaN | No | NaN |
| Mesoplasma\_florum\_L1 | **Path** | B | 1 | 0 | - | Facultative | Host-associated | NaN | Mammal, | NaN |
| Mesorhizobium\_BNC1 | **Free** | B | 1 | 3 | - | Aerobic | Multiple | NaN | No | NaN |
| Methylacidiphilum\_infernorum\_V4 | **Free** | B | 1 | 0 | NaN | Aerobic | Specialized | NaN | NaN | NaN |
| Methylibium\_petroleiphilum\_PM1 | **Free** | B | 1 | 1 | - | Facultative | Aquatic | 30 | NaN | NaN |
| Methylobacillus\_flagellatus\_KT | **Free** | B | 1 | 0 | - | Aerobic | Specialized | NaN | NaN | NaN |
| Methylobacterium\_4\_46 | **Free** | B | 1 | 2 | - | Facultative | Multiple | NaN | No | NaN |
| Methylobacterium\_chloromethanicum\_CM4 | **Free** | B | 1 | 2 | - | Aerobic | Terrestrial | 30 | NaN | NaN |
| Methylobacterium\_extorquens\_DM4 | **Free** | B | 1 | 2 | - | Aerobic | Terrestrial | NaN | NaN | NaN |
| Methylobacterium\_extorquens\_PA1 | **Free** | B | 1 | 0 | - | Facultative | Multiple | NaN | No | NaN |
| Methylobacterium\_radiotolerans\_JCM\_2831 | **Path** | B | 1 | 8 | NaN | Aerobic | Host-associated | NaN | No | None |
| Methylobacterium\_radiotolerans\_JCM\_2831 | **Path** | B | 1 | 8 | NaN | Aerobic | Host-associated | NaN | No | None |
| Methylocella\_silvestris\_BL2 | **Free** | B | 1 | 0 | - | Aerobic | Terrestrial | NaN | No | NaN |
| Methylotenera\_mobilis\_JLW8 | **Free** | B | 1 | 0 | - | Aerobic | Aquatic | NaN | No | NaN |
| Methylovorus\_SIP3\_4 | **Free** | B | 1 | 2 | - | Aerobic | Aquatic | NaN | No | NaN |
| Methylovorus\_SIP3\_4 | **Free** | B | 1 | 2 | - | Aerobic | Aquatic | NaN | No | NaN |
| Micrococcus\_luteus\_NCTC\_2665 | **Free** | B | 1 | 0 | + | Aerobic | Multiple | NaN | NaN | NaN |
| Microcystis\_aeruginosa\_NIES\_843 | **Path** | B | 1 | 0 | NaN | Aerobic | Aquatic | NaN | Animal, | Cyanobacterial |
| Moorella\_thermoacetica\_ATCC\_39073 | **Free** | B | 1 | 0 | + | Anaerobic | Aquatic | 58 | NaN | NaN |
| Mycobacterium\_JLS | **Free** | B | 1 | 0 | + | NaN | Multiple | NaN | No | None |
| Mycobacterium\_KMS | **Free** | B | 1 | 2 | + | NaN | Multiple | NaN | No | None |
| Mycobacterium\_abscessus\_ATCC\_19977T | **Path** | B | 1 | 1 | + | Aerobic | Multiple | 37 | Human | Lung, |
| Mycobacterium\_bovis\_BCG\_Pasteur\_1173P2 | **Path** | B | 1 | 0 | + | Aerobic | Host-associated | NaN | Bovine | Bovine |
| Mycobacterium\_bovis\_BCG\_Pasteur\_1173P2 | **Path** | B | 1 | 0 | + | Aerobic | Host-associated | NaN | Bovine | Bovine |
| Mycobacterium\_bovis\_BCG\_Pasteur\_1173P2 | **Path** | B | 1 | 0 | + | Aerobic | Host-associated | NaN | Bovine | Bovine |
| Mycobacterium\_bovis\_BCG\_Tokyo\_172 | **Path** | B | 1 | 0 | + | Aerobic | Host-associated | NaN | Human | NaN |
| Mycobacterium\_leprae\_Br4923 | **Path** | B | 1 | 0 | + | Aerobic | Host-associated | 37 | Human | Leprosy |
| Mycobacterium\_marinum\_M | **Path** | B | 1 | 1 | + | Aerobic | Multiple | 32 | Fish, | Tuberculosis-like |
| Mycobacterium\_tuberculosis\_F11 | **Path** | B | 1 | 0 | + | Aerobic | Host-associated | 37 | Human | Tuberculosis |
| Mycobacterium\_tuberculosis\_H37Ra | **Path** | B | 1 | 0 | + | Aerobic | Host-associated | 37 | Human | Tuberculosis |
| Mycobacterium\_tuberculosis\_H37Rv | **Path** | B | 1 | 0 | + | Aerobic | Host-associated | 37 | Human | Tuberculosis |
| Mycobacterium\_tuberculosis\_KZN\_1435 | **Path** | B | 1 | 0 | + | Aerobic | Host-associated | 37 | Human | Tuberculosis |
| Mycobacterium\_ulcerans\_Agy99 | **Path** | B | 1 | 1 | + | Aerobic | Host-associated | 32 | Human | Buruli |
| Mycoplasma\_agalactiae\_PG2 | **Path** | B | 1 | 0 | - | Facultative | Host-associated | NaN | Sheep, | Mastitis, |
| Mycoplasma\_arthritidis\_158L3\_1 | **Path** | B | 1 | 0 | - | Facultative | Host-associated | 37 | Rodent | Acute |
| Mycoplasma\_capricolum\_ATCC\_27343 | **Path** | B | 1 | 0 | - | Facultative | Host-associated | 37 | Ruminant | Severe |
| Mycoplasma\_conjunctivae | **Path** | B | 1 | 0 | NaN | Microaerophilic | Host-associated | NaN | Sheep, | infectious |
| Mycoplasma\_hyopneumoniae\_232 | **Path** | B | 1 | 0 | - | Facultative | Host-associated | 37 | Swine | Swine |
| Mycoplasma\_hyopneumoniae\_7448 | **Path** | B | 1 | 0 | - | Facultative | Host-associated | 37 | Swine | Enzootic |
| Mycoplasma\_hyopneumoniae\_J | **Path** | B | 1 | 0 | - | Facultative | Host-associated | 37 | Swine | Enzootic |
| Mycoplasma\_mycoides | **Path** | B | 1 | 0 | - | Facultative | Host-associated | 37 | Cattle | Contagious |
| Mycoplasma\_synoviae\_53 | **Path** | B | 1 | 0 | - | Facultative | Host-associated | 37 | Chicken | Chronic |
| Myxococcus\_xanthus\_DK\_1622 | **Free** | B | 1 | 0 | - | Aerobic | Terrestrial | NaN | No | None |
| Nakamurella\_multipartita\_DSM\_44233 | **Free** | B | 1 | 0 | + | NaN | Terrestrial | NaN | No | NaN |
| Natranaerobius\_thermophilus\_JW\_NM\_WN\_LF | **Free** | B | 1 | 2 | + | NaN | Specialized | NaN | No | NaN |
| Nautilia\_profundicola\_AmH | **Free** | B | 1 | 0 | - | Anaerobic | Multiple | 45 | NaN | NaN |
| Neisseria\_gonorrhoeae\_NCCP11945 | **Path** | B | 1 | 1 | - | Aerobic | Host-associated | NaN | Human | Gonorrhea |
| Neisseria\_meningitidis\_053442 | **Path** | B | 1 | 0 | - | Aerobic | Host-associated | NaN | Human | Meningitis |
| Neisseria\_meningitidis\_053442 | **Path** | B | 1 | 0 | - | Aerobic | Host-associated | NaN | Human | Meningitis |
| Neisseria\_meningitidis\_053442 | **Path** | B | 1 | 0 | - | Aerobic | Host-associated | NaN | Human | Meningitis |
| Neisseria\_meningitidis\_053442 | **Path** | B | 1 | 0 | - | Aerobic | Host-associated | NaN | Human | Meningitis |
| Neisseria\_meningitidis\_053442 | **Path** | B | 1 | 0 | - | Aerobic | Host-associated | NaN | Human | Meningitis |
| Neisseria\_meningitidis\_053442 | **Path** | B | 1 | 0 | - | Aerobic | Host-associated | NaN | Human | Meningitis |
| Neisseria\_meningitidis\_053442 | **Path** | B | 1 | 0 | - | Aerobic | Host-associated | NaN | Human | Meningitis |
| Neisseria\_meningitidis\_053442 | **Path** | B | 1 | 0 | - | Aerobic | Host-associated | NaN | Human | Meningitis |
| Neorickettsia\_risticii\_Illinois | **Path** | B | 1 | 0 | NaN | NaN | Host-associated | NaN | Horse | Potomac |
| Neorickettsia\_sennetsu\_Miyayama | **Path** | B | 1 | 0 | NaN | NaN | Multiple | NaN | Human | Sennetsu |
| Nitratiruptor\_SB155-2 | **Free** | B | 1 | 0 | - | Anaerobic | Specialized | NaN | NaN | NaN |
| Nitrobacter\_hamburgensis\_X14 | **Free** | B | 1 | 3 | - | Aerobic | Terrestrial | NaN | No | NaN |
| Nitrobacter\_winogradskyi\_Nb-255 | **Free** | B | 1 | 0 | - | Facultative | Terrestrial | NaN | No | NaN |
| Nitrosococcus\_oceani\_ATCC\_19707 | **Free** | B | 1 | 1 | NaN | NaN | Aquatic | NaN | NaN | NaN |
| Nitrosomonas\_eutropha\_C71 | **Free** | B | 1 | 2 | - | NaN | Multiple | NaN | NaN | NaN |
| Nitrosospira\_multiformis\_ATCC\_25196 | **Free** | B | 1 | 3 | - | Aerobic | Terrestrial | NaN | NaN | NaN |
| Nocardioides\_JS614 | **Free** | B | 1 | 1 | + | Aerobic | Terrestrial | 30 | No | None |
| Nostoc\_sp | **Free** | B | 1 | 6 | NaN | Aerobic | Multiple | NaN | No | NaN |
| Ochrobactrum\_anthropi\_ATCC\_49188 | **Path** | B | 2 | 4 | NaN | NaN | Terrestrial | NaN | Human | Opportunistic |
| Oligotropha\_carboxidovorans\_OM5 | **Free** | B | 1 | 0 | - | NaN | Multiple | NaN | No | NaN |
| Onion\_yellows\_phytoplasma | **Path** | B | 1 | 0 | NaN | Aerobic | Host-associated | NaN | Onion | Onions |
| Opitutus\_terrae\_PB90\_1 | **Free** | B | 1 | 0 | NaN | Anaerobic | Aquatic | NaN | No | NaN |
| Orientia\_tsutsugamushi\_Boryong | **Path** | B | 1 | 0 | - | NaN | Host-associated | NaN | Human | Scrub |
| Orientia\_tsutsugamushi\_Ikeda | **Path** | B | 1 | 0 | - | NaN | Host-associated | NaN | Human | Scrub |
| Paenibacillus\_JDR\_2 | **Free** | B | 1 | 0 | + | Aerobic | Terrestrial | NaN | No | NaN |
| Parabacteroides\_distasonis\_ATCC\_8503 | **Path** | B | 1 | 0 | + | Anaerobic | Host-associated | NaN | Mammal | Opportunistic |
| Parachlamydia\_sp\_UWE25 | **Path** | B | 1 | 0 | - | NaN | Host-associated | NaN | Animal | Probable |
| Paracoccus\_denitrificans\_PD1222 | **Free** | B | 2 | 1 | - | Aerobic | Multiple | NaN | No | NaN |
| Parvibaculum\_lavamentivorans\_DS-1 | **Free** | B | 1 | 0 | - | Aerobic | Multiple | NaN | No | NaN |
| Pectobacterium\_wasabiae\_WPP163 | **Path** | B | 1 | 0 | NaN | NaN | Multiple | NaN | plants | soft |
| Pedobacter\_heparinus\_DSM\_2366 | **Free** | B | 1 | 0 | - | Aerobic | Terrestrial | NaN | No | NaN |
| Pelobacter\_carbinolicus | **Free** | B | 1 | 0 | - | Anaerobic | Aquatic | NaN | No | NaN |
| Pelobacter\_propionicus\_DSM\_2379 | **Free** | B | 1 | 2 | - | Anaerobic | Multiple | 30 | No | None |
| Chlorobium\_luteolum\_DSM\_273 | **Free** | B | 1 | 0 | - | Anaerobic | Multiple | 25 | No | NaN |
| Pelodictyon\_phaeoclathratiforme\_BU\_1 | **Free** | B | 1 | 0 | - | Anaerobic | Multiple | NaN | No | NaN |
| Pelotomaculum\_thermopropionicum\_SI | **Free** | B | 1 | 0 | NaN | Anaerobic | Specialized | 55 | No | None |
| Persephonella\_marina\_EX\_H1 | **Free** | B | 1 | 1 | - | Microaerophilic | Aquatic | 73 | No | None |
| Petrotoga\_mobilis\_SJ95 | **Free** | B | 1 | 0 | - | Anaerobic | Specialized | NaN | No | NaN |
| Phenylobacterium\_zucineum\_HLK1 | **Path** | B | 1 | 1 | - | Aerobic | Host-associated | 37 | Human | NaN |
| Photobacterium\_profundum\_SS9 | **Free** | B | 2 | 1 | - | Facultative | Multiple | 15 | No | NaN |
| Photorhabdus\_luminescens | **Path** | B | 1 | 0 | - | Facultative | Host-associated | NaN | Insect | Toxemia |
| Polaromonas\_JS666 | **Free** | B | 1 | 2 | - | Aerobic | Multiple | 20 | No | None |
| Polaromonas\_naphthalenivorans\_CJ2 | **Free** | B | 1 | 8 | - | Aerobic | Aquatic | 20 | No | NaN |
| Polynucleobacter\_necessarius\_asymbioticus\_QLW\_P1DMWA\_1 | **Free** | B | 1 | 0 | - | Aerobic | Aquatic | NaN | NaN | NaN |
| Porphyromonas\_gingivalis\_ATCC\_33277 | **Path** | B | 1 | 0 | - | Anaerobic | Host-associated | 37 | Human | Periodontal |
| Prochlorococcus\_marinus\_AS9601 | **Path** | B | 1 | 0 | - | NaN | Aquatic | NaN | No | None |
| Prochlorococcus\_marinus\_AS9601 | **Path** | B | 1 | 0 | - | NaN | Aquatic | NaN | No | None |
| Prochlorococcus\_marinus\_CCMP1375 | **Free** | B | 1 | 0 | NaN | NaN | Aquatic | NaN | NaN | NaN |
| Prochlorococcus\_marinus\_MED4 | **Free** | B | 1 | 0 | NaN | NaN | Aquatic | NaN | NaN | NaN |
| Prochlorococcus\_marinus\_MIT\_9211 | **Free** | B | 1 | 0 | - | NaN | Aquatic | NaN | No | None |
| Prochlorococcus\_marinus\_MIT\_9215 | **Free** | B | 1 | 0 | - | NaN | Aquatic | NaN | No | None |
| Prochlorococcus\_marinus\_MIT\_9301 | **Free** | B | 1 | 0 | NaN | NaN | Aquatic | NaN | NaN | NaN |
| Prochlorococcus\_marinus\_MIT\_9301 | **Free** | B | 1 | 0 | NaN | NaN | Aquatic | NaN | NaN | NaN |
| Prochlorococcus\_marinus\_MIT\_9303 | **Free** | B | 1 | 0 | - | NaN | Aquatic | NaN | No | None |
| Prochlorococcus\_marinus\_MIT\_9312 | **Free** | B | 1 | 0 | - | NaN | Aquatic | NaN | NaN | NaN |
| Prochlorococcus\_marinus\_MIT\_9515 | **Free** | B | 1 | 0 | NaN | NaN | Aquatic | NaN | No | None |
| Prochlorococcus\_marinus\_NATL1A | **Free** | B | 1 | 0 | - | NaN | Aquatic | NaN | No | NaN |
| Prochlorococcus\_marinus\_NATL2A | **Free** | B | 1 | 0 | - | NaN | Aquatic | NaN | NaN | NaN |
| Propionibacterium\_acnes\_KPA171202 | **Path** | B | 1 | 0 | + | Anaerobic | Host-associated | 37 | Human | Acne |
| Propionibacterium\_acnes\_KPA171202 | **Path** | B | 1 | 0 | + | Anaerobic | Host-associated | 37 | Human | Acne |
| Propionibacterium\_acnes\_KPA171202 | **Path** | B | 1 | 0 | + | Anaerobic | Host-associated | 37 | Human | Acne |
| Propionibacterium\_acnes\_KPA171202 | **Path** | B | 1 | 0 | + | Anaerobic | Host-associated | 37 | Human | Acne |
| Prosthecochloris\_aestuarii\_DSM\_271 | **Free** | B | 1 | 1 | - | NaN | Aquatic | 30 | No | None |
| Prosthecochloris\_vibrioformis\_DSM\_265 | **Free** | B | 1 | 0 | - | Facultative | Aquatic | NaN | No | None |
| Proteus\_mirabilis | **Path** | B | 1 | 1 | - | Aerobic | Host-associated | 37 | Human | Encephalitis, |
| Pseudoalteromonas\_atlantica\_T6c | **Path** | B | 1 | 0 | - | Aerobic | Aquatic | NaN | Shellfish | Shell |
| Pseudoalteromonas\_haloplanktis\_TAC125 | **Free** | B | 2 | 0 | - | Aerobic | Aquatic | NaN | No | NaN |
| Pseudomonas\_aeruginosa\_LESB58 | **Path** | B | 1 | 0 | - | Aerobic | Multiple | NaN | Human | Lung |
| Pseudomonas\_aeruginosa\_LESB58 | **Path** | B | 1 | 0 | - | Aerobic | Multiple | NaN | Human | Lung |
| Pseudomonas\_aeruginosa\_LESB58 | **Path** | B | 1 | 0 | - | Aerobic | Multiple | NaN | Human | Lung |
| Pseudomonas\_aeruginosa\_LESB58 | **Path** | B | 1 | 0 | - | Aerobic | Multiple | NaN | Human | Lung |
| Pseudomonas\_aeruginosa\_PA7 | **Path** | B | 1 | 0 | - | Aerobic | Multiple | NaN | Human | Opportunistic |
| Pseudomonas\_entomophila\_L48 | **Path** | B | 1 | 0 | NaN | NaN | Multiple | NaN | Insect | Cellular |
| Pseudomonas\_fluorescens\_SBW25 | **Free** | B | 1 | 1 | - | Aerobic | Multiple | NaN | NaN | NaN |
| Pseudomonas\_mendocina\_ymp | **Path** | B | 1 | 0 | - | Aerobic | Multiple | NaN | Human | Spondylodiscitis |
| Pseudomonas\_putida\_F1 | **Free** | B | 1 | 0 | - | Aerobic | Multiple | NaN | No | None |
| Pseudomonas\_putida\_GB\_1 | **Free** | B | 1 | 0 | - | Aerobic | Multiple | NaN | No | None |
| Pseudomonas\_putida\_GB\_1 | **Free** | B | 1 | 0 | - | Aerobic | Multiple | NaN | No | None |
| Pseudomonas\_putida\_W619 | **Free** | B | 1 | 0 | - | Aerobic | Multiple | NaN | No | None |
| Pseudomonas\_syringae\_phaseolicola\_1448A | **Path** | B | 1 | 2 | - | Aerobic | Multiple | NaN | Plant | Plant |
| Psychrobacter\_PRwf-1 | **Free** | B | 1 | 2 | - | Aerobic | Aquatic | NaN | NaN | NaN |
| Psychrobacter\_arcticum\_273-4 | **Free** | B | 1 | 0 | - | NaN | Specialized | 22 | No | NaN |
| Psychrobacter\_cryohalolentis\_K5 | **Free** | B | 1 | 1 | NaN | NaN | Multiple | NaN | NaN | NaN |
| Psychromonas\_ingrahamii\_37 | **Free** | B | 1 | 0 | - | Anaerobic | Aquatic | NaN | No | NaN |
| Ralstonia\_eutropha\_H16 | **Path** | B | 2 | 1 | - | Facultative | Specialized | 30 | NaN | NaN |
| Ralstonia\_eutropha\_H16 | **Path** | B | 2 | 1 | - | Facultative | Specialized | 30 | NaN | NaN |
| Ralstonia\_eutropha\_JMP134 | **Free** | B | 2 | 2 | NaN | Facultative | Multiple | 30 | NaN | NaN |
| Ralstonia\_pickettii\_12D | **Free** | B | 2 | 3 | - | Aerobic | Multiple | NaN | No | NaN |
| Ralstonia\_pickettii\_12D | **Free** | B | 2 | 3 | - | Aerobic | Multiple | NaN | No | NaN |
| Ralstonia\_pickettii\_12J | **Free** | B | 2 | 1 | - | Aerobic | Multiple | NaN | NaN | NaN |
| Renibacterium\_salmoninarum\_ATCC\_33209 | **Path** | B | 1 | 0 | + | Facultative | Host-associated | 15 | Salmonid | Bacterial |
| Renibacterium\_salmoninarum\_ATCC\_33209 | **Path** | B | 1 | 0 | + | Facultative | Host-associated | 15 | Salmonid | Bacterial |
| Rhizobium\_leguminosarum\_bv\_viciae\_3841 | **Path** | B | 1 | 6 | - | Aerobic | Host-associated | NaN | No | NaN |
| Rhizobium\_leguminosarum\_bv\_viciae\_3841 | **Path** | B | 1 | 6 | - | Aerobic | Host-associated | NaN | No | NaN |
| Rhodobacter\_sphaeroides\_ATCC\_17025 | **Free** | B | 1 | 5 | - | Facultative | Multiple | NaN | No | NaN |
| Rhodobacter\_sphaeroides\_ATCC\_17029 | **Free** | B | 2 | 1 | - | Facultative | Multiple | NaN | No | NaN |
| Rhodobacter\_sphaeroides\_KD131 | **Free** | B | 2 | 2 | - | Facultative | Multiple | NaN | NaN | NaN |
| Rhodococcus\_jostii\_RHA1 | **Free** | B | 1 | 3 | + | Aerobic | Terrestrial | 30 | NaN | NaN |
| Rhodococcus\_opacus\_B4 | **Free** | B | NaN | 0 | + | Aerobic | Terrestrial | NaN | NaN | NaN |
| Rhodococcus\_opacus\_B4 | **Free** | B | NaN | 0 | + | Aerobic | Terrestrial | NaN | NaN | NaN |
| Rhodococcus\_opacus\_B4 | **Free** | B | NaN | 0 | + | Aerobic | Terrestrial | NaN | NaN | NaN |
| Rhodoferax\_ferrireducens\_T118 | **Free** | B | 1 | 1 | - | Facultative | Multiple | 25 | No | NaN |
| Rhodopseudomonas\_palustris\_BisA53 | **Free** | B | 1 | 0 | - | Facultative | Multiple | NaN | No | NaN |
| Rhodopseudomonas\_palustris\_BisB18 | **Free** | B | 1 | 0 | - | Facultative | Multiple | NaN | No | NaN |
| Rhodopseudomonas\_palustris\_BisB5 | **Free** | B | 1 | 0 | - | Facultative | Multiple | NaN | No | NaN |
| Rhodopseudomonas\_palustris\_HaA2 | **Free** | B | 1 | 0 | - | Facultative | Multiple | NaN | No | NaN |
| Rhodospirillum\_centenum\_SW | **Free** | B | 1 | 0 | - | Facultative | Aquatic | NaN | NaN | NaN |
| Rhodothermus\_marinus\_DSM\_4252 | **Free** | B | 1 | 1 | - | Aerobic | Specialized | 65 | No | NaN |
| Rickettsia\_africae\_ESF\_5 | **Path** | B | 1 | 1 | - | Aerobic | Host-associated | NaN | Human | African |
| Rickettsia\_akari\_Hartford | **Path** | B | 1 | 0 | - | Aerobic | Host-associated | NaN | Human | Rickettsialpox |
| Rickettsia\_bellii\_RML369-C | **Path** | B | 1 | 0 | - | NaN | Host-associated | NaN | NaN | NaN |
| Rickettsia\_bellii\_RML369-C | **Path** | B | 1 | 0 | - | NaN | Host-associated | NaN | NaN | NaN |
| Rickettsia\_bellii\_RML369-C | **Path** | B | 1 | 0 | - | NaN | Host-associated | NaN | NaN | NaN |
| Rickettsia\_canadensis\_McKiel | **Path** | B | 1 | 0 | - | Aerobic | Host-associated | NaN | Human | Epidemic |
| Rickettsia\_felis\_URRWXCal2 | **Path** | B | 1 | 2 | - | NaN | Host-associated | NaN | Human, | Spotted-fever |
| Rickettsia\_felis\_URRWXCal2 | **Path** | B | 1 | 2 | - | NaN | Host-associated | NaN | Human, | Spotted-fever |
| Rickettsia\_rickettsii\_Iowa | **Path** | B | 1 | 0 | - | Aerobic | Host-associated | NaN | Human | Rocky |
| Rickettsia\_rickettsii\_Sheila\_Smith | **Path** | B | 1 | 0 | - | Aerobic | Host-associated | 37 | Human | Rocky |
| Rickettsia\_typhi\_wilmington | **Path** | B | 1 | 0 | - | Aerobic | Host-associated | NaN | Human, | Endemic |
| Robiginitalea\_biformata\_HTCC2501 | **Free** | B | 1 | 0 | - | Aerobic | Aquatic | 30 | No | NaN |
| Roseiflexus\_RS-1 | **Free** | B | 1 | 0 | - | Facultative | Specialized | NaN | NaN | NaN |
| Roseiflexus\_castenholzii\_DSM\_13941 | **Free** | B | 1 | 0 | NaN | Facultative | Aquatic | 50 | No | NaN |
| Roseobacter\_denitrificans\_OCh\_114 | **Free** | B | 1 | 4 | - | NaN | Multiple | NaN | No | NaN |
| Rubrobacter\_xylanophilus\_DSM\_9941 | **Free** | B | 1 | 0 | + | Aerobic | Specialized | 60 | No | None |
| Saccharomonospora\_viridis\_DSM\_43017 | **Path** | B | 1 | 0 | NaN | NaN | Multiple | 37 | Human | Farmer |
| Saccharophagus\_degradans\_2-40 | **Free** | B | 1 | 0 | - | Aerobic | Aquatic | NaN | No | NaN |
| Saccharopolyspora\_erythraea\_NRRL\_2338 | **Free** | B | 1 | 0 | + | Aerobic | Terrestrial | NaN | No | NaN |
| Salinibacter\_ruber\_DSM\_13855 | **Free** | B | 1 | 1 | - | Aerobic | Specialized | NaN | No | None |
| Salinispora\_arenicola\_CNS-205 | **Free** | B | 1 | 0 | + | Aerobic | Aquatic | NaN | No | None |
| Salinispora\_tropica\_CNB-440 | **Free** | B | 1 | 0 | + | Aerobic | Aquatic | 28 | No | None |
| Salmonella\_enterica\_Choleraesuis | **Path** | B | 1 | 2 | - | Facultative | Host-associated | 37 | Human, | Salmonellosis |
| Salmonella\_enterica\_Paratypi\_ATCC\_9150 | **Path** | B | 1 | 0 | - | Facultative | Host-associated | 37 | Human | Paratyphoid |
| Salmonella\_enterica\_arizonae\_serovar\_62\_z4\_z23\_\_ | **Path** | B | 1 | 0 | - | Facultative | Host-associated | NaN | Human, | Gastroenteritis |
| Salmonella\_enterica\_serovar\_Agona\_SL483 | **Path** | B | 1 | 1 | - | Facultative | Multiple | NaN | Human, | Gastroenteritis |
| Salmonella\_enterica\_serovar\_Dublin\_CT\_02021853 | **Path** | B | 1 | 1 | - | Facultative | Multiple | NaN | Human, | Bacteremia, |
| Salmonella\_enterica\_serovar\_Enteritidis\_P125109 | **Path** | B | 1 | 0 | - | Facultative | Specialized | NaN | Human, | Salmonellosis |
| Salmonella\_enterica\_serovar\_Gallinarum\_287\_91 | **Path** | B | 1 | 0 | - | Facultative | Multiple | NaN | Poultry | Fowl |
| Salmonella\_enterica\_serovar\_Heidelberg\_SL476 | **Path** | B | 1 | 2 | - | Facultative | Multiple | NaN | Human, | Gastroenteritis |
| Salmonella\_enterica\_serovar\_Newport\_SL254 | **Path** | B | 1 | 2 | - | Facultative | Multiple | NaN | Human, | Gastroenteritis |
| Salmonella\_enterica\_serovar\_Paratyphi\_A\_AKU\_12601 | **Path** | B | 1 | 0 | - | Facultative | Multiple | NaN | Human | Paratyphoid |
| Salmonella\_enterica\_serovar\_Paratyphi\_B\_SPB7 | **Path** | B | 1 | 0 | - | Facultative | Host-associated | 37 | Human | Typhoid-like |
| Salmonella\_enterica\_serovar\_Paratyphi\_C\_RKS4594 | **Path** | B | 1 | 1 | - | Facultative | Host-associated | 37 | Human | Paratyphoid |
| Salmonella\_enterica\_serovar\_Schwarzengrund\_CVM19633 | **Path** | B | 1 | 2 | - | Facultative | Multiple | NaN | Human, | Gastroenteritis |
| Serratia\_proteamaculans\_568 | **Path** | B | 1 | 1 | NaN | Facultative | Multiple | NaN | Human | Pneumonia |
| Shewanella\_ANA-3 | **Free** | B | 1 | 1 | - | Facultative | Multiple | NaN | NaN | NaN |
| Shewanella\_MR-4 | **Free** | B | 1 | 0 | - | Facultative | Multiple | NaN | NaN | NaN |
| Shewanella\_MR-7 | **Free** | B | 1 | 1 | - | Facultative | Aquatic | NaN | NaN | NaN |
| Shewanella\_W3-18-1 | **Path** | B | 1 | 0 | - | Facultative | Multiple | NaN | Human | Soft |
| Shewanella\_amazonensis\_SB2B | **Free** | B | 1 | 0 | - | Facultative | Multiple | 37 | NaN | NaN |
| Shewanella\_baltica\_OS155 | **Free** | B | 1 | 4 | - | Facultative | Aquatic | NaN | NaN | NaN |
| Shewanella\_baltica\_OS155 | **Free** | B | 1 | 4 | - | Facultative | Aquatic | NaN | NaN | NaN |
| Shewanella\_baltica\_OS185 | **Free** | B | 1 | 1 | - | Facultative | Aquatic | NaN | No | None |
| Shewanella\_baltica\_OS195 | **Free** | B | 1 | 3 | - | Facultative | Aquatic | NaN | NaN | NaN |
| Shewanella\_baltica\_OS223 | **Free** | B | 1 | 3 | - | Facultative | Aquatic | NaN | No | NaN |
| Shewanella\_denitrificans\_OS217 | **Free** | B | 1 | 0 | - | Facultative | Aquatic | NaN | NaN | NaN |
| Shewanella\_frigidimarina\_NCIMB\_400 | **Free** | B | 1 | 0 | - | Facultative | Multiple | NaN | NaN | NaN |
| Shewanella\_loihica\_PV-4 | **Free** | B | 1 | 0 | - | Facultative | Multiple | NaN | NaN | NaN |
| Shewanella\_piezotolerans\_WP3 | **Free** | B | 1 | 0 | - | Facultative | Specialized | NaN | No | None |
| Shewanella\_putrefaciens\_CN-32 | **Path** | B | 1 | 0 | - | Facultative | Multiple | NaN | Human | Soft |
| Shewanella\_sediminis\_HAW-EB3 | **Free** | B | 1 | 0 | - | Facultative | Aquatic | 10 | No | NaN |
| Shewanella\_woodyi\_ATCC\_51908 | **Free** | B | 1 | 0 | - | Facultative | Multiple | 25 | No | None |
| Shigella\_boydii\_CDC\_3083\_94 | **Path** | B | 1 | 5 | - | Facultative | Host-associated | 37 | Human | Dysentery |
| Shigella\_boydii\_Sb227 | **Path** | B | 1 | 1 | - | Facultative | Host-associated | 37 | Human | Dysentery |
| Shigella\_dysenteriae | **Path** | B | 1 | 2 | - | Facultative | Host-associated | 37 | Human | Dysentery |
| Shigella\_dysenteriae | **Path** | B | 1 | 2 | - | Facultative | Host-associated | 37 | Human | Dysentery |
| Shigella\_dysenteriae | **Path** | B | 1 | 2 | - | Facultative | Host-associated | 37 | Human | Dysentery |
| Shigella\_dysenteriae | **Path** | B | 1 | 2 | - | Facultative | Host-associated | 37 | Human | Dysentery |
| Shigella\_dysenteriae | **Path** | B | 1 | 2 | - | Facultative | Host-associated | 37 | Human | Dysentery |
| Shigella\_dysenteriae | **Path** | B | 1 | 2 | - | Facultative | Host-associated | 37 | Human | Dysentery |
| Shigella\_dysenteriae | **Path** | B | 1 | 2 | - | Facultative | Host-associated | 37 | Human | Dysentery |
| Shigella\_flexneri\_2a | **Path** | B | 1 | 1 | - | Facultative | Host-associated | 37 | Human | Dysentery |
| Shigella\_flexneri\_2a\_2457T | **Path** | B | 1 | 0 | - | Facultative | Host-associated | 37 | Human | Dysentery |
| Shigella\_flexneri\_5\_8401 | **Path** | B | 1 | 0 | - | Facultative | Host-associated | 37 | Human | Dysentery |
| Shigella\_sonnei\_Ss046 | **Path** | B | 1 | 4 | - | Facultative | Host-associated | 37 | Human | Dysentery |
| Silicibacter\_TM1040 | **Free** | B | 1 | 2 | - | NaN | Multiple | NaN | No | NaN |
| Sinorhizobium\_medicae\_WSM419 | **Free** | B | 1 | 3 | - | Aerobic | Multiple | 28 | No | None |
| Slackia\_heliotrinireducens\_DSM\_20476 | **Free** | B | 1 | 0 | + | Anaerobic | Multiple | NaN | No | NaN |
| Solibacter\_usitatus\_Ellin6076 | **Free** | B | 1 | 0 | - | Aerobic | Terrestrial | 30 | No | None |
| Sorangium\_cellulosum\_\_So\_ce\_56\_ | **Free** | B | 1 | 0 | - | Aerobic | Terrestrial | NaN | No | NaN |
| Sphingomonas\_wittichii\_RW1 | **Free** | B | 1 | 2 | - | Aerobic | Aquatic | NaN | No | NaN |
| Sphingopyxis\_alaskensis\_RB2256 | **Free** | B | 1 | 1 | - | Aerobic | Aquatic | NaN | NaN | NaN |
| Staphylococcus\_aureus\_JH1 | **Path** | B | 1 | 1 | + | Facultative | Host-associated | NaN | Human, | Toxic-shock |
| Staphylococcus\_aureus\_JH9 | **Path** | B | 1 | 1 | + | Facultative | Host-associated | NaN | Human, | Toxic-shock |
| Staphylococcus\_aureus\_Mu3 | **Path** | B | 1 | 0 | + | Facultative | Host-associated | NaN | Human | Variety |
| Staphylococcus\_aureus\_N315 | **Free** | B | 1 | 1 | + | Facultative | Host-associated | NaN | Human | Toxic-shock |
| Staphylococcus\_aureus\_Newman | **Path** | B | 1 | 0 | + | NaN | NaN | NaN | Human, | Skin |
| Staphylococcus\_aureus\_USA300\_FPR3757 | **Path** | B | 1 | 3 | + | Facultative | Host-associated | 37 | Human | Septicemia, |
| Staphylococcus\_aureus\_USA300\_TCH1516 | **Path** | B | 1 | 2 | + | Facultative | Host-associated | 37 | Human | Septicemia, |
| Staphylococcus\_epidermidis\_ATCC\_12228 | **Path** | B | 1 | 6 | + | Facultative | Host-associated | NaN | Human | Toxic-shock |
| Staphylococcus\_epidermidis\_ATCC\_12228 | **Path** | B | 1 | 6 | + | Facultative | Host-associated | NaN | Human | Toxic-shock |
| Staphylococcus\_haemolyticus | **Path** | B | 1 | 3 | + | Facultative | Host-associated | NaN | Human | Wide |
| Staphylococcus\_haemolyticus | **Path** | B | 1 | 3 | + | Facultative | Host-associated | NaN | Human | Wide |
| Staphylococcus\_saprophyticus | **Path** | B | 1 | 2 | + | Aerobic | Host-associated | NaN | Human | Urinary |
| Stenotrophomonas\_maltophilia\_K279a | **Path** | B | 1 | 0 | - | Aerobic | Multiple | NaN | Human | Blood, |
| Stenotrophomonas\_maltophilia\_R551\_3 | **Free** | B | 1 | 0 | - | Aerobic | Multiple | NaN | NaN | NaN |
| Streptobacillus\_moniliformis\_DSM\_12112 | **Path** | B | 1 | 1 | - | Microaerophilic | Host-associated | NaN | Human | Rat |
| Streptococcus\_dysgalactiae\_equisimilis\_GGS\_124 | **Path** | B | 1 | 0 | + | NaN | Host-associated | NaN | Human | Endocarditis |
| Streptococcus\_dysgalactiae\_equisimilis\_GGS\_124 | **Path** | B | 1 | 0 | + | NaN | Host-associated | NaN | Human | Endocarditis |
| Streptococcus\_dysgalactiae\_equisimilis\_GGS\_124 | **Path** | B | 1 | 0 | + | NaN | Host-associated | NaN | Human | Endocarditis |
| Streptococcus\_dysgalactiae\_equisimilis\_GGS\_124 | **Path** | B | 1 | 0 | + | NaN | Host-associated | NaN | Human | Endocarditis |
| Streptococcus\_dysgalactiae\_equisimilis\_GGS\_124 | **Path** | B | 1 | 0 | + | NaN | Host-associated | NaN | Human | Endocarditis |
| Streptococcus\_dysgalactiae\_equisimilis\_GGS\_124 | **Path** | B | 1 | 0 | + | NaN | Host-associated | NaN | Human | Endocarditis |
| Streptococcus\_dysgalactiae\_equisimilis\_GGS\_124 | **Path** | B | 1 | 0 | + | NaN | Host-associated | NaN | Human | Endocarditis |
| Streptococcus\_dysgalactiae\_equisimilis\_GGS\_124 | **Path** | B | 1 | 0 | + | NaN | Host-associated | NaN | Human | Endocarditis |
| Streptococcus\_dysgalactiae\_equisimilis\_GGS\_124 | **Path** | B | 1 | 0 | + | NaN | Host-associated | NaN | Human | Endocarditis |
| Streptococcus\_equi\_4047 | **Free** | B | 1 | 0 | + | Facultative | Host-associated | NaN | Equine | Strangles |
| Streptococcus\_equi\_4047 | **Free** | B | 1 | 0 | + | Facultative | Host-associated | NaN | Equine | Strangles |
| Streptococcus\_equi\_zooepidemicus | **Path** | B | 1 | 0 | + | Facultative | Host-associated | NaN | Animals, | Inflammatory |
| Streptococcus\_equi\_zooepidemicus\_MGCS10565 | **Path** | B | 1 | 0 | + | Facultative | Host-associated | NaN | Animals, | Opportunistic |
| Streptococcus\_pneumoniae\_70585 | **Path** | B | 1 | 0 | NaN | Facultative | Multiple | NaN | Human | Pneumonia |
| Streptococcus\_pneumoniae\_CGSP14 | **Path** | B | 1 | 0 | + | Facultative | Multiple | NaN | Human | Pneumonia |
| Streptococcus\_pneumoniae\_D39 | **Path** | B | 1 | 0 | + | Facultative | Multiple | NaN | Human | Pneumonia |
| Streptococcus\_pneumoniae\_G54 | **Path** | B | 1 | 0 | + | Facultative | Host-associated | NaN | Human | Pneumonia |
| Streptococcus\_pneumoniae\_Hungary19A\_6 | **Path** | B | 1 | 0 | + | Facultative | Multiple | NaN | Human | Pneumonia |
| Streptococcus\_pneumoniae\_JJA | **Path** | B | 1 | 0 | + | Facultative | Multiple | NaN | Human | Pneumonia |
| Streptococcus\_pneumoniae\_P1031 | **Path** | B | 1 | 0 | - | Facultative | Multiple | NaN | Human | Pneumonia |
| Streptococcus\_pneumoniae\_Taiwan19F\_14 | **Path** | B | 1 | 0 | + | Facultative | Multiple | NaN | Human | Pneumonia |
| Streptococcus\_pyogenes\_MGAS10270 | **Path** | B | 1 | 0 | + | Facultative | Host-associated | NaN | Human | Wide |
| Streptococcus\_pyogenes\_MGAS10394 | **Path** | B | 1 | 0 | + | Facultative | Host-associated | 35 | Human | Wide |
| Streptococcus\_pyogenes\_MGAS10750 | **Path** | B | 1 | 0 | + | Facultative | Host-associated | NaN | Human | Wide |
| Streptococcus\_pyogenes\_MGAS2096 | **Path** | B | 1 | 0 | + | Facultative | Host-associated | NaN | Human | Wide |
| Streptococcus\_pyogenes\_MGAS5005 | **Path** | B | 1 | 0 | + | Facultative | Host-associated | 35 | Human | Wide |
| Streptococcus\_pyogenes\_MGAS6180 | **Path** | B | 1 | 0 | + | Facultative | Host-associated | 35 | Human | Wide |
| Streptococcus\_pyogenes\_MGAS9429 | **Path** | B | 1 | 0 | + | Facultative | Host-associated | NaN | Human | Wide |
| Streptococcus\_pyogenes\_Manfredo | **Path** | B | 1 | 0 | + | Facultative | Host-associated | NaN | Human | Wide |
| Streptococcus\_pyogenes\_NZ131 | **Path** | B | 1 | 0 | + | Facultative | Host-associated | NaN | Human | Wide |
| Streptococcus\_sanguinis\_SK36 | **Path** | B | 1 | 0 | + | Facultative | Host-associated | NaN | Human | Endocarditis |
| Streptococcus\_suis\_05ZYH33 | **Path** | B | 1 | 0 | + | Facultative | Multiple | 37 | Swine, | Meningitis, |
| Streptococcus\_suis\_98HAH33 | **Path** | B | 1 | 0 | + | Facultative | Specialized | 37 | Swine, | Meningitis, |
| Streptococcus\_suis\_BM407 | **Path** | B | 1 | 1 | + | Facultative | Multiple | NaN | Swine, | Meningitis, |
| Streptococcus\_suis\_BM407 | **Path** | B | 1 | 1 | + | Facultative | Multiple | NaN | Swine, | Meningitis, |
| Streptococcus\_suis\_P1\_7 | **Path** | B | 1 | 0 | + | Facultative | Multiple | NaN | Swine, | Meningitis, |
| Streptococcus\_suis\_SC84 | **Path** | B | 1 | 0 | + | Facultative | Multiple | NaN | Swine, | Meningitis, |
| Streptococcus\_thermophilus\_CNRZ1066 | **Free** | B | 1 | 0 | + | Anaerobic | Multiple | 45 | No | NaN |
| Streptococcus\_thermophilus\_LMD-9 | **Free** | B | 1 | 2 | + | Facultative | Multiple | 37 | No | None |
| Streptococcus\_thermophilus\_LMG\_18311 | **Free** | B | 1 | 0 | + | Anaerobic | Multiple | 45 | No | NaN |
| Streptococcus\_uberis\_0140J | **Path** | B | 1 | 0 | + | Facultative | Multiple | 37 | Cattle | Mastitis |
| Streptomyces\_griseus\_NBRC\_13350 | **Free** | B | 1 | 0 | + | Aerobic | Multiple | NaN | No | None |
| Streptosporangium\_roseum\_DSM\_43021 | **Free** | B | 1 | 1 | + | Aerobic | Terrestrial | NaN | No | NaN |
| Sulfurihydrogenibium\_YO3AOP1 | **Free** | B | 1 | 0 | - | Facultative | Specialized | NaN | No | NaN |
| Sulfurihydrogenibium\_azorense\_Az\_Fu1 | **Path** | B | 1 | 0 | - | Microaerophilic | Aquatic | 68 | No | None |
| Sulfurihydrogenibium\_azorense\_Az\_Fu1 | **Path** | B | 1 | 0 | - | Microaerophilic | Aquatic | 68 | No | None |
| Sulfurihydrogenibium\_azorense\_Az\_Fu1 | **Path** | B | 1 | 0 | - | Microaerophilic | Aquatic | 68 | No | None |
| Sulfurospirillum\_deleyianum\_DSM\_6946 | **Free** | B | 1 | 0 | - | Microaerophilic | Aquatic | NaN | No | NaN |
| Sulfurovum\_NBC37-1 | **Free** | B | 1 | 0 | - | Facultative | Specialized | NaN | No | None |
| Symbiobacterium\_thermophilum\_IAM14863 | **Free** | B | 1 | 0 | + | Microaerophilic | Terrestrial | 60 | No | None |
| Synechococcus\_CC9311 | **Free** | B | 1 | 0 | NaN | NaN | Aquatic | NaN | No | NaN |
| Synechococcus\_CC9605 | **Free** | B | 1 | 0 | NaN | NaN | Aquatic | NaN | No | NaN |
| Synechococcus\_CC9902 | **Free** | B | 1 | 0 | NaN | NaN | Aquatic | NaN | No | NaN |
| Synechococcus\_PCC\_7002 | **Free** | B | 1 | 6 | NaN | NaN | Aquatic | 38 | No | NaN |
| Synechococcus\_RCC307 | **Free** | B | 1 | 0 | NaN | NaN | Aquatic | NaN | NaN | NaN |
| Synechococcus\_WH\_7803 | **Free** | B | 1 | 0 | NaN | NaN | Aquatic | NaN | NaN | NaN |
| Synechococcus\_elongatus\_PCC\_6301 | **Free** | B | 1 | 0 | - | NaN | Aquatic | NaN | No | None |
| Synechococcus\_elongatus\_PCC\_7942 | **Free** | B | 1 | 1 | NaN | NaN | Aquatic | NaN | NaN | NaN |
| Synechococcus\_sp\_WH8102 | **Free** | B | 1 | 0 | NaN | NaN | Aquatic | NaN | No | NaN |
| Syntrophobacter\_fumaroxidans\_MPOB | **Free** | B | 1 | 0 | - | Anaerobic | Aquatic | 37 | No | NaN |
| Syntrophomonas\_wolfei\_Goettingen | **Free** | B | 1 | 0 | - | Anaerobic | Multiple | NaN | No | NaN |
| Syntrophus\_aciditrophicus\_SB | **Free** | B | 1 | 0 | - | Anaerobic | Multiple | 35 | NaN | NaN |
| Thauera\_MZ1T | **Free** | B | 1 | 1 | - | Facultative | Aquatic | NaN | No | None |
| Thermanaerovibrio\_acidaminovorans\_DSM\_6589 | **Free** | B | 1 | 0 | - | Anaerobic | Terrestrial | 55 | No | NaN |
| Thermoanaerobacter\_pseudethanolicus\_ATCC\_33223 | **Free** | B | 1 | 0 | + | Anaerobic | Aquatic | NaN | No | NaN |
| Thermoanaerobacter\_tengcongensis | **Free** | B | 1 | 0 | - | Anaerobic | Specialized | 75 | No | NaN |
| Thermobaculum\_terrenum\_ATCC\_BAA\_798 | **Free** | B | 2 | 0 | + | Aerobic | Specialized | 67 | No | NaN |
| Thermodesulfovibrio\_yellowstonii\_DSM\_11347 | **Free** | B | 1 | 0 | - | NaN | Specialized | 65 | No | NaN |
| Thermofilum\_pendens\_Hrk\_5 | **Path** | A | 1 | 1 | NaN | Anaerobic | Specialized | 88 | No | NaN |
| Thermomicrobium\_roseum\_DSM\_5159 | **Free** | B | 1 | 1 | - | Aerobic | Specialized | 70 | No | NaN |
| Thermomicrobium\_roseum\_DSM\_5159 | **Free** | B | 1 | 1 | - | Aerobic | Specialized | 70 | No | NaN |
| Thermomicrobium\_roseum\_DSM\_5159 | **Free** | B | 1 | 1 | - | Aerobic | Specialized | 70 | No | NaN |
| Thermomonospora\_curvata\_DSM\_43183 | **Free** | B | 1 | 0 | + | Aerobic | Specialized | NaN | No | NaN |
| Thermosipho\_africanus\_TCF52B | **Free** | B | 1 | 0 | - | Anaerobic | Specialized | 75 | No | NaN |
| Thermotoga\_RQ2 | **Free** | B | 1 | 0 | - | Anaerobic | Specialized | NaN | No | NaN |
| Thermotoga\_lettingae\_TMO | **Free** | B | 1 | 0 | - | Anaerobic | Specialized | 65 | No | NaN |
| Thermotoga\_lettingae\_TMO | **Free** | B | 1 | 0 | - | Anaerobic | Specialized | 65 | No | NaN |
| Thermotoga\_neapolitana\_DSM\_4359 | **Free** | B | 1 | 0 | - | Microaerophilic | Specialized | NaN | NaN | NaN |
| Thermotoga\_petrophila\_RKU-1 | **Free** | B | 1 | 0 | - | Anaerobic | Aquatic | 80 | No | NaN |
| Thermus\_thermophilus\_HB27 | **Free** | B | 1 | 1 | NaN | Aerobic | Specialized | 68 | NaN | NaN |
| Thermus\_thermophilus\_HB8 | **Free** | B | 1 | 2 | NaN | Aerobic | Specialized | NaN | NaN | NaN |
| Thioalkalivibrio\_HL\_EbGR7 | **Free** | B | 1 | 0 | - | Aerobic | Specialized | NaN | No | NaN |
| Thiobacillus\_denitrificans\_ATCC\_25259 | **Free** | B | 1 | 0 | - | Facultative | Multiple | NaN | No | NaN |
| Thiomicrospira\_crunogena\_XCL-2 | **Free** | B | 1 | 0 | - | Microaerophilic | Aquatic | NaN | No | NaN |
| Tolumonas\_auensis\_DSM\_9187 | **Free** | B | 1 | 0 | - | Facultative | Aquatic | 22 | No | NaN |
| Treponema\_pallidum\_SS14 | **Path** | B | 1 | 0 | NaN | Anaerobic | Host-associated | NaN | Human | Syphilis |
| Trichodesmium\_erythraeum\_IMS101 | **Free** | B | 1 | 0 | NaN | Aerobic | Aquatic | NaN | No | NaN |
| Ureaplasma\_parvum\_serovar\_3\_ATCC\_27815 | **Path** | B | 1 | 0 | NaN | Facultative | Host-associated | NaN | Human, | Urogenital |
| Ureaplasma\_urealyticum\_serovar\_10\_ATCC\_33699 | **Path** | B | 1 | 0 | + | Facultative | Host-associated | 37 | Human | Urogenital |
| Variovorax\_paradoxus\_S110 | **Free** | B | 2 | 0 | - | Aerobic | Multiple | NaN | No | NaN |
| Veillonella\_parvula\_DSM\_2008 | **Path** | B | 1 | 0 | - | Anaerobic | Host-associated | NaN | Human | rare |
| Vibrio\_cholerae\_M66\_2 | **Path** | B | 2 | 0 | - | Facultative | Multiple | NaN | Human | Cholera |
| Vibrio\_cholerae\_MJ\_1236 | **Path** | B | 2 | 0 | - | Facultative | Aquatic | NaN | Human | Cholera |
| Vibrio\_cholerae\_MJ\_1236 | **Path** | B | 2 | 0 | - | Facultative | Aquatic | NaN | Human | Cholera |
| Vibrio\_cholerae\_MJ\_1236 | **Path** | B | 2 | 0 | - | Facultative | Aquatic | NaN | Human | Cholera |
| Vibrio\_cholerae\_MJ\_1236 | **Path** | B | 2 | 0 | - | Facultative | Aquatic | NaN | Human | Cholera |
| Vibrio\_cholerae\_O395 | **Path** | B | 2 | 0 | - | Facultative | Aquatic | NaN | Human, | Cholera |
| Vibrio\_fischeri\_ES114 | **Free** | B | 2 | 1 | - | Facultative | Multiple | NaN | No | None |
| Vibrio\_fischeri\_MJ11 | **Free** | B | 2 | 1 | - | Facultative | Multiple | NaN | No | None |
| Vibrio\_harveyi\_ATCC\_BAA-1116 | **Path** | B | 2 | 1 | - | Facultative | Aquatic | NaN | Vertebrate | Vibriosis |
| Vibrio\_splendidus\_LGP32 | **Path** | B | 2 | 0 | - | Facultative | Aquatic | NaN | Fish, | Vibriosis |
| Vibrio\_vulnificus\_CMCP6 | **Path** | B | 2 | 0 | - | Facultative | Aquatic | NaN | Human | Gastroenteritis, |
| Vibrio\_vulnificus\_YJ016 | **Path** | B | 2 | 1 | - | Facultative | Aquatic | NaN | Human | Gastroenteritis, |
| Wolbachia\_endosymbiont\_of\_Culex\_quinquefasciatus\_Pel | **Path** | B | 1 | 0 | - | Aerobic | Host-associated | NaN | No | NaN |
| Xanthobacter\_autotrophicus\_Py2 | **Free** | B | 1 | 1 | - | Facultative | Multiple | NaN | No | NaN |
| Xanthomonas\_campestris\_vesicatoria\_85-10 | **Path** | B | 1 | 4 | - | Aerobic | Host-associated | NaN | Plant | Bacterial |
| Xanthomonas\_citri | **Path** | B | 1 | 2 | - | Aerobic | Host-associated | NaN | Plant | Citrus |
| Xanthomonas\_oryzae\_KACC10331 | **Path** | B | 1 | 0 | - | Aerobic | Host-associated | NaN | Rice | Rice |
| Xanthomonas\_oryzae\_MAFF\_311018 | **Path** | B | 1 | 0 | - | Aerobic | Host-associated | NaN | Rice | Rice |
| Xanthomonas\_oryzae\_PXO99A | **Path** | B | 1 | 0 | - | Aerobic | Host-associated | NaN | Rice | Rice |
| Xylanimonas\_cellulosilytica\_DSM\_15894 | **Free** | B | 1 | 1 | + | Aerobic | Specialized | NaN | NaN | NaN |
| Xylella\_fastidiosa\_M12 | **Path** | B | 1 | 0 | - | Aerobic | Host-associated | NaN | Citrus | Citrus |
| Yersinia\_pestis\_Angola | **Path** | B | 1 | 2 | - | Facultative | Multiple | NaN | Human, | Bubonic |
| Yersinia\_pestis\_Antiqua | **Path** | B | 1 | 3 | - | Facultative | Multiple | NaN | Human, | Bubonic |
| Yersinia\_pestis\_Nepal516 | **Path** | B | 1 | 2 | - | Facultative | Multiple | NaN | Human, | Bubonic |
| Yersinia\_pestis\_Pestoides\_F | **Path** | B | 1 | 2 | - | Facultative | Multiple | NaN | Human, | Bubonic |
| Yersinia\_pestis\_biovar\_Microtus\_91001 | **Path** | B | 1 | 4 | - | Facultative | Multiple | NaN | Human, | Bubonic |
| Yersinia\_pseudotuberculosis\_IP32953 | **Path** | B | 1 | 2 | - | Facultative | Multiple | NaN | Human, | Gastroenteritis |
| Yersinia\_pseudotuberculosis\_IP\_31758 | **Path** | B | 1 | 2 | - | Facultative | Multiple | NaN | Human, | Gastroenteritis |
| Yersinia\_pseudotuberculosis\_IP\_31758 | **Path** | B | 1 | 2 | - | Facultative | Multiple | NaN | Human, | Gastroenteritis |
| Yersinia\_pseudotuberculosis\_IP\_31758 | **Path** | B | 1 | 2 | - | Facultative | Multiple | NaN | Human, | Gastroenteritis |
| Yersinia\_pseudotuberculosis\_PB1\_ | **Path** | B | 1 | 1 | - | Facultative | Multiple | NaN | Human | Gastroenteritis |
| Yersinia\_pseudotuberculosis\_YPIII | **Path** | B | 1 | 0 | - | Facultative | Multiple | NaN | Human | Gastroenteritis |
| Zymomonas\_mobilis\_ZM4 | **Free** | B | 1 | 0 | - | Anaerobic | Multiple | NaN | No | NaN |
